# Red Seaweed-Derived Phycobiliproteins: Marine Bioactive Colorants with Functional Health Properties

**DOI:** 10.3390/md24070246

**Published:** 2026-07-15

**Authors:** Yiming Sun, Yi Zhou, Minyao Wang, Faezeh Ebrahimi, Muhammad Sajid Arshad, Colin J. Barrow, Hafiz Ansar Rasul Suleria

**Affiliations:** 1 School of Agriculture, Food and Ecosystem Sciences, Faculty of Science, The University of Melbourne, Parkville, VIC 3010, Australia; 2 Centre for Sustainable Bioproducts, School of Life and Environmental Sciences, Deakin University, Waurn Ponds, VIC 3216, Australia

**Keywords:** phycobiliproteins, red seaweeds, natural colorants, clean-label foods, functional ingredients, sustainable food systems

## Abstract

Color is a critical sensory attribute of foods that strongly influences consumer perception, acceptance, and purchasing decisions. As health-oriented consumption increases, natural pigments are progressively replacing synthetic colorants. Red algal phycobiliproteins (PBPs) have gained attention in related industries for their vivid water-soluble colors and potential health-promoting properties. Phycoerythrin (PE), phycocyanin (PC), and allophycocyanin (APC) are key pigment proteins responsible for their characteristic coloration. However, variations in algal species, season, and cultivation environments make PBP composition, yield, and quality difficult to standardize. This review summarizes red algal PBPs, covering algal sources, extraction and purification, physicochemical properties, biological functions, regulatory frameworks, and future directions. Major red seaweed sources, PBP types, and extraction strategies are compared, with cyanobacterial PBP studies incorporated where direct red algal evidence is limited. Evidence suggests that red algal PBPs are promising for clean-label, water-based, and mildly processed foods, including beverages, dairy products, confectionery, and meat alternatives, but their application is constrained by sensitivity to heat, light, oxygen, and pH. Beyond coloration, PBPs exhibit antioxidant, anti-inflammatory, anticancer, antibacterial, and metabolic health-promoting activities. Overall, red algal PBPs have considerable potential as dual-function ingredients, although commercialization requires advances in raw material standardization, stability-enhancement strategies, process optimization, clinical validation, and regulatory harmonization.

## 1. Introduction

In the food industry, color is a key visual attribute that strongly influences consumers’ purchasing decisions and perceived product quality. Within the food processing sector, increasing emphasis has been placed on developing visually appealing product coloration [1]. Although synthetic colorants offer high stability and low cost, these advantages no longer fully align with the preferences of modern consumers. This shift is driven by a growing demand for healthier lifestyles and concerns regarding the potential environmental and health impacts of synthetic colorants [2]. At the same time, consumer demand for “clean label” and “naturally sourced” products has directly encouraged the development of natural colorants. An increasing number of companies are seeking to replace synthetic colorants with natural alternatives, while also aiming for these pigments to provide not only appealing colors but also evidence-based functional benefits, such as antioxidant and anti-inflammatory activities, thereby supporting product positioning related to “functionality” and “healthiness” [3]. However, in practical applications, natural colorants often suffer from issues such as fading, discoloration, and loss of bioactivity due to variations in pH, heat treatment, light exposure, and oxidation. For instance, elevated storage and processing temperatures can accelerate the degradation of anthocyanins, while extreme pH conditions or the presence of metal ions can reduce the stability of carotenoids [4]. Although limitations in stability and scalability remain key bottlenecks for industrial application [5], the shift toward natural colorants continues to accelerate across the food industry.

Among various natural colorants, Phycobiliproteins (PBPs) are considered promising candidates for “next-generation natural colorants” due to their strong coloring capacity, vivid color range, and excellent water solubility. PBPs are the constituent proteins of phycobilisomes; their apoproteins and chromophores are covalently linked via thioether bonds to form functional pigment-protein complexes [6]. The presence of these chromophores endows PBPs with strong absorption and fluorescence properties in the visible light region. Compared with natural colorants such as anthocyanins, betanin, and carotenoids, PBPs exhibit a more vivid and “pure” coloration, are readily dispersible in aqueous systems, and are therefore well suited for applications in modern food formulations. In particular, PBPs can provide bright red to blue hues and are among the few natural pigments capable of serving as viable alternatives for achieving blue and green tones in foods [7]. Recent reviews have focused on the sustainable production of PBPs and their applications in food and health-related fields, systematically summarizing progress in extraction and purification techniques, processing stability, biofunctional activities, and industrialization pathways.

PBPs are a class of pigment-protein complexes found in photosynthetic organisms and can be classified according to their absorption characteristics into phycoerythrin (PE), phycocyanin (PC), and allophycocyanin (APC) [6,8]. Red seaweeds are among the important natural sources of PBPs, being particularly rich in R-phycoerythrin (R-PE), APC, and other pigment proteins [9]. These proteins exhibit a color range from pink to orange-red and may also demonstrate notable antioxidant activity in certain systems [8]. Red seaweed materials are widely available and include edible red algae such as *Porphyra* and *Pyropia*, as well as agar-producing species such as *Gracilaria* and *Gelidium* [10,11]. However, their pigment content and composition are influenced by species differences, seasonal variation, light conditions, and cultivation practices, which in turn affect extraction yield, purity, and color characteristics [6,12].

It is worth noting that red algal colorants may serve not only as “coloring agents” but also as potential functional ingredients with health-related benefits. In addition to their coloring properties, PBPs have been widely reported to exhibit multiple biological activities, including reactive oxygen species (ROS) scavenging, alleviation of oxidative stress, modulation of inflammatory mediators and related signaling pathways, inhibition of abnormal cell proliferation, and induction of apoptosis (primarily supported by in vitro evidence), as well as antibacterial and antiviral activities; they may also contribute to the regulation of metabolic disorders [8,13,14]. Although current evidence is largely derived from in vitro and animal studies, with limited clinical and human data available, PBPs nonetheless demonstrate a clear dual advantage as both edible colorants and potential bioactive compounds.

This review was prepared as a narrative review rather than a systematic review. The relevant literature was collected from major academic databases and publisher platforms, including Web of Science, PubMed, ScienceDirect, Google Scholar, and relevant journal websites. The search was guided by combinations of keywords related to red algae, red seaweed, Rhodophyta, phycobiliproteins, phycoerythrin, phycocyanin, extraction, purification, stability, natural colorants, food applications, bioactivities, safety, and regulation. To reflect recent progress in this field, the review primarily included studies published within the last 10 years. A small number of earlier studies were retained only when they provided essential background information on PBP structure, extraction, purification, or regulatory issues. Papers were included when they were directly relevant to the scope of this review, especially red algal PBPs and their potential use as natural food colorants and functional bioactive ingredients.

Unlike previous reviews that broadly address PBPs across cyanobacteria, microalgae, or general pigment applications, this review specifically focuses on red seaweed-derived PBPs as marine bioactive colorants for food systems. Selected studies on PBPs from cyanobacteria or other non-red-seaweed sources are discussed only where direct evidence from red seaweeds is limited, particularly in relation to bioactivity, food applications, stabilization strategies, and regulatory considerations. These studies are included as supportive references where they provide relevant insights into PBP properties and potential applications, but are not treated as direct evidence for red seaweed-derived PBPs. Accordingly, it synthesizes existing evidence within a “source–process–performance–function” framework. We outline the primary sources and types of red algal PBPs; compare conventional and emerging green extraction and purification approaches, along with their commercial feasibility; summarize their color performance in representative food systems and the key factors governing stability; and critically evaluate reported bioactivities and potential health benefits based on clearly defined levels of evidence. Furthermore, this paper summarizes formulation strategies and representative application cases that integrate color functionality with bioactivity and discusses safety considerations, regulatory frameworks, consumer acceptance, and sustainability issues, as well as current research gaps, commercialization pathways, and future perspectives.

## 2. Sources and Types of Phycobiliproteins

PBPs are water-soluble multimeric proteins that play an important role in light energy harvesting in cyanobacteria and many red algae [8]. Structurally, PBPs consist of polypeptide chains covalently bound to open-chain tetrapyrrole chromophores (phycobilins). The major phycobilins include phycoerythrobilin (PEB), phycourobilin (PUB), phycocyanobilin (PCB), and phycoviolobilin/cryptoviolobilin (CVB) [15]. These phycobilins are responsible for the strong visible-light absorption and fluorescence properties of PBPs, which underpin their physiological roles and make them valuable for various industrial applications [16].

**Figure 1:**
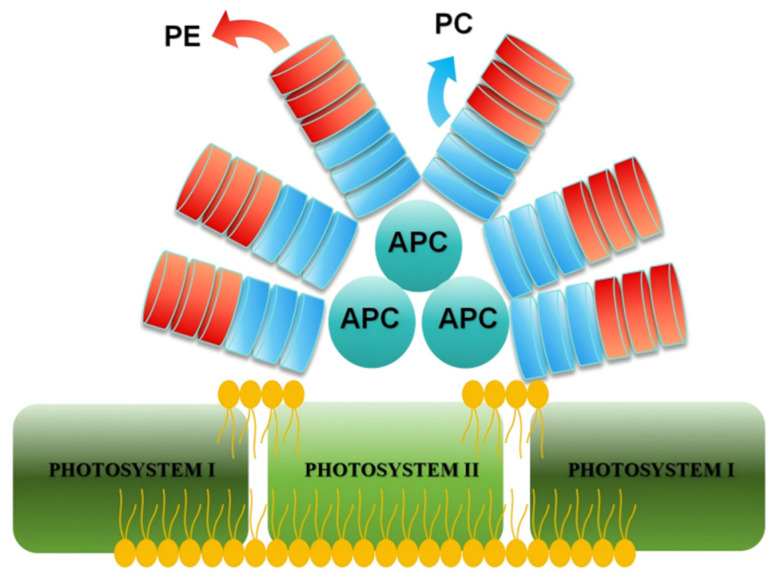
Schematic structure of Phycobilisomes. Abbreviations: PE, phycoerythrin; PC, phycocyanin; APC, allophycocyanin. Schematic created by the authors with reference to previously published phycobilisome diagrams [18,19].

Under physiological conditions, PBPs are not randomly distributed but are assembled with linker proteins into large supramolecular complexes known as phycobilisomes (PBSs), which are typically attached to the stromal side of thylakoid membranes [17]. PBSs consist of two distinct structural components: the rods and the core. The rods are composed of two to three types of PBPs, with PC located proximal to the core, while PE and phycoerythrocyanin (PEC) are positioned toward the distal ends. The core comprises three cylinders, each approximately 12–15 nm in length, with each cylinder exhibiting a triangular cross-section and containing four APC trimers [16,18]. PBSs function as light-harvesting antenna complexes that efficiently absorb light in the wavelength range of approximately 450–650 nm and transfer the captured energy to the photosynthetic reaction centers located in the thylakoid membrane [17]. The general structure of phycobilisomes, including the spatial arrangement of PE, PC, and APC, is illustrated in Figure 1.

### 2.1. PBPs in Red Seaweed: R-PE, R-Phycocyanin (R-PC), and APC

**Figure 2:**
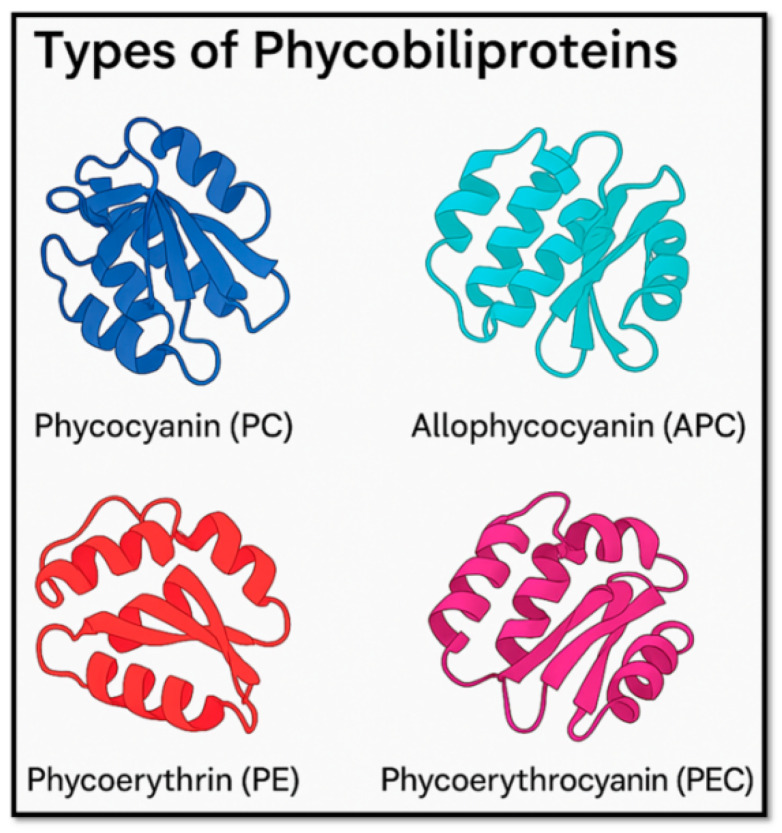
Structure and classification of phycobiliproteins. Reprinted from “Phycobilins Versatile Pigments with Wide-Ranging Applications: Exploring Their Uses, Biological Activities, Extraction Methods and Future Perspectives” by Garcia-Gomez, Aguirre-Cavazos, Chavez-Montes, Ballesteros-Torres, Orozco-Flores, Reyna-Martinez, Torres-Hernandez, Gonzalez-Meza, Castillo-Hernandez, Gloria-Garza, Kacaniova, Ireneusz-Kluz and Elizondo-Luevano [14], published in Marine Drugs under the Creative Commons Attribution (CC BY 4.0) license.

PBPs are generally classified into three main categories based on their spectral characteristics: PE, PC, and APC. However, recent studies have proposed PEC as a fourth category [14,18]. While some reviews describe PEC as an alternative form of PE found in certain cyanobacteria, its pigment composition places it spectrally between PE and PC; therefore, it may be considered either as a distinct class or as a related intermediate form within the broader PBP family [16,20]. PE exhibits considerable diversity in chromophore composition, subunit organization, and spectral properties. It can exist in multiple oligomeric states, including trimers (α_3_β_3_), hexamers ((α_3_β_3_)_2_), and other higher-order assemblies, with chromophores covalently attached to the protein complex. PC is typically composed of α and β subunits, with chromophores attached at specific cysteine residues, including α-84 on the α subunit and β-84 and β-155 on the β subunit. APC is generally a more structurally conserved protein, consisting of α and β subunits, each carrying a single phycobilin chromophore (α-84 and β-84) [18,21]. On this basis, different lineages and pigment compositions give rise to distinct subtypes; for example, PE includes R-PE, B-PE, and C-PE, while PC includes R-PC and C-phycocyanin (C-PC) [21]. The prefixes reflect the original source organism from which these pigments were first isolated: R denotes Rhodophytan, C denotes cyanobacterial, and B denotes Bangiophycean origin [14,18]. Importantly, these prefixes are not merely historical labels; they also have scientific significance, as they often correlate with lineage-specific structural variations and distinct spectral properties. The structural organization and major classification of PBPs are summarized in Figure 2.

To improve clarity and consistency in PBP terminology, the main abbreviations and subtype nomenclature used in this review are summarized in Table 1.

In red seaweed, the most representative and common types are R-PE, R-PC, and APC.

#### 2.1.1. R-PE

The main source of R-PE is red algae (e.g., the genera *Porphyra* and *Gracilaria*), where it not only predominates in abundance but also represents one of the most spectrally distinctive PBPs [26]. PE subunits are highly abundant within the phycobilisome (PBS) structure of red algae, and evidence suggests that PE can account for approximately 80% of the total PBPs in *Porphyridium purpureum* [26,27]. These pigments are also responsible for the characteristic reddish-purple coloration and associated color variations observed in red seaweeds [28]. Structurally, R-PE is composed of multiple subunits assembled into a hexameric complex. It typically contains PEB and PUB chromophores covalently attached via thioether linkages. Specifically, two PEB molecules are attached to the α subunit, while the β subunit carries additional PEB and PUB chromophores, with PUB commonly linked via a cysteine residue in a thioether bond [8]. Some studies indicate that PE subunits predominantly bind PEB, enabling strong absorption in the 500–560 nm range (green light). In many marine species, however, PUB is also present, which enhances the absorption of blue light and provides an adaptive advantage in underwater light environments [29,30]. Functionally, PE is located at the distal end of the peripheral rods of the PBS, where it serves as the primary light-harvesting component for high-energy photons. It initiates a directional energy transfer cascade, passing excitation energy inward through PC and ultimately to APC in the core [16].

#### 2.1.2. R-PC

R-PC is frequently found in red seaweeds and exhibits a distinct blue to violet coloration with potential applications as a natural colorant. Reported data indicate that the PE and PC contents in *Pyropia haitanensis* (formerly *Porphyra haitanensis*) range from 1.499–8.882 mg/g and 1.402–7.634 mg/g, respectively, demonstrating that red algae can accumulate substantial amounts of PC [31]. R-PC can be classified based on spectral characteristics into R-PC(I), R-PC(II), and R-PC(III), all of which contain chromophores at the α84, β84, and β155 sites [22]. More recent studies have further proposed R-PC(IV) and R-PC(V), which differ in their chromophore-binding sites compared with the first three types [14]. Importantly, these subtypes differ in the specific phycobilins they bind, resulting in distinct spectral properties and pigmentation profiles. In PBS, R-PC is typically located in rod structures closer to the core. Its absorption maximum occurs at longer wavelengths than that of R-PE, consistent with the directional energy transfer gradient, where it functions as a spectral intermediary between PE and APC [16]. A recent study on purified *Porphyra* spp. (nori) reported that R-PC exhibits high purity (A618/A280 ≥ 3.4), predominantly α-helical secondary structure (~53%), and a trimeric (αβ)_3_ configuration in solution, consistent with its native conformation [32]. These properties support its potential application as a natural food colorant; however, limitations related to processing stability have also been identified. For instance, exposure to 60 °C can cause irreversible losses in both color and antioxidant activity, while its stability is generally higher under acidic conditions than under alkaline conditions [32].

#### 2.1.3. APC

The content of APC in red algae is generally second only to that of R-PC and R-PE. Zhang, Ma, Liu, Qin, Sun, Zhao, and Sui [25] analyzed the PBS supercomplex structure of *Griffithsia pacifica* using single-particle cryo-electron microscopy and modeled a total of 48 APC subunits in the core region. This energy-transfer hub comprises three subtypes: allophycocyanin-B (APC-B), APC core-membrane linker (APC-Lcm), and APC. The two basal cylinders are formed by APC-B and APC-Lcm, whereas the core cylinder is primarily composed of APC [21].

### 2.2. Species Distribution

From a taxonomic perspective, PBPs are widely distributed among red algae; however, the relative proportions of R-PE, R-PC, and APC vary substantially among species and habitats, reflecting differences in ecological niches and light-adaptation strategies.

Several reviews on the biochemical composition of red algae indicate that PBPs constitute a significant fraction of total algal proteins, accounting for approximately 50% of soluble proteins and up to 20% of dry algal biomass. Within the pigment-protein pool, R-PE and R-PC are often the predominant components [33,34].

In edible red algae such as *Pyropia* and *Porphyra*, R-PE is often regarded as a key endogenous pigment and a primary target for extraction. For instance, *Neopyropia yezoensis* (previously known as *Pyropia yezoensis*) is frequently used as a model organism in studies on R-PE separation and purification and has been reported to exhibit notable cytotoxic effects on HepG2 cells, suggesting potential anticancer bioactivity derived from the source alga [35]. In addition, a study on *Pyropia haitanensis* (previously known as *Porphyra haitanensis*) reported phycoerythrin, phycocyanin, and APC contents of 1.499–8.882 mg/g, 1.402–7.634 mg/g, and 0.315–1.623 mg/g, respectively [31]. Similarly, other red algae used in the colloid industry, such as agarophytes (*Gracilaria* and *Gelidium*), also possess typical PBS systems composed of PE, PC, and APC; however, the relative proportions of these pigments vary considerably depending on environmental conditions and developmental stage [17,36]. Previous studies on *Gracilaria gracilis* have clearly demonstrated seasonal variation in PBP concentrations, with R-PE decreasing from 7 to 3.6 mg/g dry weight (highest in winter and lowest in autumn), APC decreasing from 3.5 to 1.5 mg/g, and PC decreasing from 3 to 0.7 mg/g (the lowest among the three) [37]. Consistent patterns have also been reported in other studies, where the relative abundance follows the trend R-PE (7 mg/g) > APC (3.5 mg/g) > PC (2 mg/g) [38].

In another red alga, *Gelidium amansii*, the R-PE content is reported to be 53 μg/g, and the R-PC content is 56 μg/g, indicating nearly equivalent levels of the two pigments. Consequently, compared with *Gracilaria*, the PBP composition pattern in *Gelidium* is markedly different, while APC content is less frequently reported in this species [39]. It should also be noted that classical physiological studies suggest that PBS heterogeneity may occur even within a single species; for example, different PBS morphologies have been reported in *Porphyra umbilicalis*. This indicates that species-level distribution patterns should be interpreted as probabilistic tendencies rather than fixed characteristics [40]. The representative literature data on PBP yields and purity indices across red seaweed species are summarized in Table 2.

### 2.3. Seasonal and Cultivation Factors Influencing PBP Extraction Yield

A consistent conclusion in studies on the physiology and resource utilization of red algae is that PBP content is highly plastic and is significantly influenced by environmental factors such as light conditions, temperature, nutrient availability, and salinity.

#### 2.3.1. Seasonality and Field Conditions

Annual monitoring of the red alga *Mastocarpus stellatus* has shown that climatic variables, including temperature, solar radiation, and precipitation, drive significant seasonal and spatial variations in phycobiliprotein (PBP) yield, with colder seasons specifically promoting increased PBP accumulation [48]. Similarly, a study on the red coralline alga *Lithothamnion glaciale* indicated that low light intensity induces pronounced adaptive changes in pigment composition and PBP content, including an approximately 10% increase in the light-harvesting capacity of PE [49]. Collectively, these findings support the conclusion that red algae may adapt to low-light and low-temperature environments by enhancing the synthesis of light-harvesting pigments. In addition, *Gracilaria domingensis* has been shown to exhibit higher pigment and protein levels in natural environments during periods of reduced solar irradiance and increased nitrogen availability [50]. This further suggests that, when PBPs are the target product, careful consideration of seasonal timing and optimal harvest windows is essential.

#### 2.3.2. Cultivation Variables

Research on the cultivation of red algae under controlled or semi-controlled conditions provides a practical basis for optimizing PBP production. The key influencing factors are generally recognized as nutrient availability, light conditions (including photoperiod and light quality), and salinity [51,52]. A study on *Gracilaria chilensis* reported that under nutrient supplementation, PC content reached 0.86 ± 0.20 and 0.89 ± 0.32 mg g^−1^ DW [53] in samples from Ancud and Chaica, respectively, whereas under non-supplemented conditions the values were reduced to 0.70 ± 0.37 and 0.73 ± 0.20 mg g^−1^ DW. A similar trend was also observed for PE. These results indicate that nutrient addition exerts a clear positive effect on PBP accumulation. In line with this, Xu et al. [54], using *Pyropia haitanensis* as the experimental model, reported that nitrogen and phosphorus deprivation negatively affects photosynthesis and pigment synthesis. The importance of light conditions has also been supported in studies on seasonal variation in PBP production. Research on *Neopyropia yezoensis* (previously known as *Pyropia yezoensis*) has shown that moderate light intensity can enhance PBP content [55]. The same study further demonstrated that interactions among salinity, light, and ocean acidification parameters (CO_2_ and pH) can jointly regulate pigment biosynthesis. These findings suggest that optimal PBP production is typically achieved under a balance between growth and photoprotection, rather than under maximal light intensity alone.

## 3. Extraction and Purification

### 3.1. Conventional Methods

The extraction of PBPs in both industrial and laboratory settings typically follows a sequential process involving the release of PBPs into the aqueous phase while minimizing denaturation and oxidation, followed by the progressive removal of co-extracted impurities such as polysaccharides, salts, phenolic compounds, lipids, and non-target proteins until the desired purity level is achieved [56,57]. Accordingly, the following sections discuss conventional PBP recovery methods according to their typical position in the downstream processing sequence, rather than as directly comparable alternatives. It should be emphasized that, although PBPs are water-soluble, the efficiency of extraction is primarily determined by the method of cellular disruption and the subsequent stepwise purification strategy, both of which critically influence yield and purity. Since approximately 70% of the total protein is located intracellularly within algal cells, conventional extraction approaches are often inefficient [58]. In addition, seaweed cell-wall polysaccharides, which form a strong gel-like structural matrix, not only hinder mechanical cell disruption but also increase system viscosity, promote ionic interactions, and further complicate downstream purification processes [56,59].

#### 3.1.1. Pretreatment and Extraction Solvent Systems

Traditional extraction methods typically employ low-temperature aqueous buffer systems, such as phosphate buffers, EDTA-containing solutions, or acetate buffers, to stabilize the chromophore–protein complexes during extraction [56]. These procedures are generally conducted under low-temperature and light-protected conditions, as multiple studies have highlighted the sensitivity of PBPs to light, heat, and pH, consistent with observations discussed in cultivation-related contexts. A recent review reported that PBPs, particularly PE, are susceptible to thermal degradation and photo-induced oxidative damage, with R-PE showing notable sensitivity to temperatures above 40 °C while remaining relatively stable within a pH range of 3–10 [15]. In addition, an experimental study assessing pigment degradation under light exposure, using the relative absorbance ratio at 565 nm (At/A0), showed that under natural light conditions, the PE sample exhibited a 10.49% decrease after 2–8 h of exposure at room temperature [60]. Overall, minimizing light exposure and maintaining low temperatures are widely recognized as essential baseline requirements in conventional PBP extraction workflows.

#### 3.1.2. Cell Disruption

Since PBPs are water-soluble chromoproteins, their extraction does not typically require as intense cell disruption as that needed for lipid-soluble pigments; however, dense algal tissues can still restrict mass transfer and reduce recovery efficiency. The most established methods include freeze–thaw cycling and low-temperature mechanical grinding. For example, in *Gracilaria chilensis*, a freeze–thaw protocol involved freezing at −20 °C for 6 h followed by thawing in the dark at room temperature for 6 h, repeated for two cycles. A similar approach was applied to *Gracilaria tenuistipitata*, although thawing was conducted at 4 °C instead [42,61]. Grinding under liquid nitrogen is also widely used. This method has been applied to *Palmaria palmata* and has likewise been used for cell disruption in *Porphyra* spp. in more recent studies, demonstrating its long-standing applicability [32,62]. Traditional wet extraction approaches are also noteworthy, typically involving soaking or hypotonic lysis. In some studies, hypotonic lysis combined with short-term mechanical refining has been used to disrupt algal cells and release intracellular proteins effectively [43]. In addition, glass bead agitation or bead milling represents a well-established and scalable mechanical disruption technique. For microalgae systems, Ruiz-Ruiz et al. [63] reported that optimal disruption conditions for *Porphyridium purpureum* (formerly *Porphyridium cruentum*) included 2900 rpm, 0.5 mm glass beads, 60% (*v*/*v*) bead loading, and a 22-min residence time. Overall, these conventional methods are technologically mature and readily scalable. However, for red algal biomass with dense tissues or a substantial polysaccharide matrix, limitations such as reduced disruption efficiency and high energy consumption remain significant challenges.

For red seaweeds, tissue grinding combined with buffer extraction is feasible; however, co-extracted polysaccharides frequently interfere with subsequent clarification and chromatographic separation processes. This limitation is widely regarded as one of the key engineering challenges in red algal extraction systems. Therefore, even when relatively mild extraction conditions are employed, robust clarification and fractionation steps are still required to obtain an extract suitable for downstream processing.

#### 3.1.3. Clarification and Concentration

After cell disruption, the traditional process described by Xu et al. [64] proceeds as follows: first, coarse filtration using gauze is performed to remove large particulate matter; this is followed by low-temperature centrifugation (4 °C) to eliminate insoluble residues, cell debris, and other impurities; finally, fine filtration is applied to further reduce suspended solids in preparation for the concentration step. These steps are also commonly adopted in conventional processing workflows. Clarification is primarily carried out to reduce suspended solids and decrease viscosity, thereby alleviating the load on downstream membrane processes. Membrane ultrafiltration (UF) is typically used for concentration. For instance, one study employed a 30 kDa polyethersulfone (PES) membrane and reported that at a volume reduction factor of 5 (VRF = 5), 100% of R-PE extracted from *Grateloupia turuturu* was recovered without denaturation, while 64.6% of polysaccharides permeated the membrane [65]. However, Novoa et al. [66] highlighted that the coexistence [46] of polysaccharides and proteins can cause severe membrane fouling and flux decline during ultrafiltration, representing a critical engineering bottleneck that must be addressed to ensure stable process operation.

#### 3.1.4. Ammonium Sulfate Precipitation and Dialysis

Ammonium sulfate precipitation is still widely used in PBP purification and is often applied for pre-enrichment and partial impurity removal prior to chromatographic separation. Several studies have reported that salting-out prior to chromatography can improve the final purity of PBPs, following a commonly adopted workflow of ammonium sulfate precipitation followed by diethylaminoethyl (DEAE) anion-exchange chromatography and Sephadex G-100 gel filtration [56,67]. Salting-out is achieved by gradually increasing the ammonium sulfate concentration to induce differential precipitation of proteins. During this process, temperature, pH, and ionic strength must be carefully controlled, as they significantly influence both precipitation behavior and the structural stability of PBPs [68,69]. Although ammonium sulfate precipitation is a classical, cost-effective, and operationally simple method, its selectivity is relatively limited, as non-target impurities may co-precipitate with PBPs, necessitating additional desalting steps prior to downstream purification.

#### 3.1.5. Chromatographic Techniques

To obtain high-purity PBPs, chromatography is widely used and extensively studied, typically involving techniques such as ion-exchange chromatography, hydroxyapatite chromatography, gel filtration, and expanded-bed adsorption chromatography [56,70]. Ion-exchange chromatography has been widely used in red algal PBP purification, including R-PE purification from *Gracilaria gracilis*, *Grateloupia turuturu*, and *Solieria filiformis* [41,44,46]. Ismail, El-Fakharany, and Hegazy [67] applied ammonium sulfate precipitation followed by sequential purification using DEAE-cellulose (anion-exchange chromatography) and Sephadex G-100 (size-exclusion chromatography). They reported that the purity index of C-PC from *Limnospira platensis* (formerly *Arthrospira platensis*) increased from 0.87 to 5.64 (A620/A280), while the purity of APC from Corallina officinalis increased from 0.49 to 5.51 (A650/A280). The anion-exchange process typically involves loading the sample under low-salt (low ionic strength) conditions, allowing the target protein to bind electrostatically to the ion-exchange resin. Subsequently, the salt concentration is gradually increased to elute bound proteins, enabling separation based on differences in charge affinity [71]. This sequential purification strategy is still regarded as a “gold standard” for achieving high purity; however, it is also among the most costly and difficult unit operations to scale up. Consequently, recent studies have focused on reducing the number of chromatographic steps or replacing certain chromatography stages with alternative separation technologies such as aqueous two-phase systems (ATPS) or membrane-based processes [72,73]. Beyond this classical route, hydroxyapatite chromatography can serve as an additional polishing option for PBP purification because its calcium phosphate matrix provides multimodal interactions different from charge-based ion-exchange chromatography or size-based gel filtration [74,75]. In large-scale R-PE purification from *Polysiphonia stricta* (formerly *Polysiphonia urceolata*), it has been used after expanded-bed adsorption as an alternative to ion-exchange chromatography, although it remains less commonly reported than ion-exchange chromatography or gel filtration [76]. Similarly, gel filtration, also known as size-exclusion chromatography, provides another polishing option by separating proteins mainly according to molecular size, and it is commonly used after initial enrichment or ion-exchange chromatography [67,77]. In contrast, expanded-bed adsorption chromatography is more suitable as an earlier capture or primary purification step before further chromatographic polishing, because it allows proteins to be recovered directly from crude or particulate-containing feedstocks [78,79].

### 3.2. Emerging Green Extraction Techniques

In recent years, research on PBP extraction has increasingly emphasized green processing approaches aimed at reducing solvent and salt consumption, lowering energy demand, shortening processing time, and minimizing potential safety and toxicity concerns, while preserving the structural and functional integrity of pigment proteins [80]. This shift has driven the development of a wide range of emerging solvents and extraction technologies that maintain extract bioactivity while integrating cell disruption, extraction, and partial purification into fewer unit operations. These innovative techniques encompass multiple processes with distinct operational principles and advantages, including enzyme-assisted extraction (EAE), ultrasound-assisted extraction (UAE), pulsed electric field (PEF), microwave-assisted extraction (MAE), pressurized liquid extraction (PLE), subcritical water extraction (SWE), supercritical fluid extraction (SFE), solid-phase extraction (SPE), osmotic lysis extraction (OL), alkaline extraction (AlE), and pH-shift extraction (pHE), among others [81,82].

#### 3.2.1. EAE

EAE employs enzyme systems composed of polysaccharide-hydrolyzing enzymes (e.g., cellulase, xylanase, and pectinase) to disrupt algal cell-wall structures, thereby enhancing the release of water-soluble PBPs [83]. A study on *Gracilaria gracilis* investigated different enzyme systems and concluded that enzyme mixtures achieved the highest extraction efficiency for freeze-dried biomass, whereas fresh samples were more suitable for single-enzyme treatments [84]. The selection of enzyme systems is generally based on the compositional characteristics of the target algal cell wall; however, composition-based selection alone may present limitations. Ghelichi et al. [85] reported that, in certain cases, polysaccharide-degrading enzymes are less effective than proteases for protein recovery, indicating that protein release mechanisms are not solely dependent on polysaccharide degradation. EAE can also be combined with emerging technologies such as ultrasound-assisted extraction. A representative recent example is the optimized extraction of wet *Grateloupia turuturu* biomass using ultrasound-assisted enzymatic hydrolysis (UAEH), which achieved a 2.3-fold higher yield compared with conventional phosphate buffer extraction after 180 min [86]. This suggests that efficient protein recovery may depend on factors beyond polysaccharide degradation alone. Overall, despite its potential, EAE still faces challenges including high cost, limited reusability, and substrate specificity. Future developments are therefore likely to focus on integrating biocatalytic approaches with physical disruption technologies to improve process sustainability and efficiency.

#### 3.2.2. UAE

UAE is one of the most commonly reported green methods for PBP recovery. UAE generates cavitation effects through ultrasound frequencies ranging from 20 kHz to 100 MHz, thereby disrupting cell structures and often significantly reducing extraction time [82]. Multiple studies have reported that UAE can increase the yield of target proteins and can also be combined with other pre-treatment methods, such as freeze–thaw cycles [82,87]. However, although UAE is a rapid and relatively energy-efficient extraction technique, its industrial-scale application remains limited. It has been reported that variations in ultrasonic power and operational parameters may induce localized hotspots, potentially leading to degradation of certain polysaccharide structures or target proteins [83]. In the literature, industrial-scale applications of UAE for PBP production remain rare, despite its theoretical potential for scale-up.

#### 3.2.3. MAE

MAE induces oscillation of polar molecules in the extraction medium through non-ionizing electromagnetic radiation (commonly in the MHz-GHz range), converting electromagnetic energy into heat and thereby disrupting cell structures and enhancing protein release [82,88]. In the PBP extraction study of *Porphyridium purpureum* conducted by Juin et al. [89], MAE was shown to significantly reduce extraction time by approximately 180- to 1080-fold compared with conventional soaking methods. However, the same study also reported a decrease in PE yield at temperatures above 40 °C, indicating that localized overheating is a critical limitation. This suggests that the primary risk associated with MAE is heat accumulation, which may lead to protein denaturation or chromophore bleaching [83]. Therefore, the application of MAE typically requires careful optimization to balance extraction efficiency with pigment-protein integrity. More recent work on the recovery of B-PE from *Porphyridium purpureum* employed MAE combined with Doehlert experimental design optimization, identifying optimal conditions of biomass/solvent ratio (16.8 mg/mL), temperature (30 °C), and extraction time (172 s); this process was further integrated with acid precipitation for purification [90]. Overall, MAE offers high extraction efficiency for PBPs but requires precise process control to minimize thermally induced degradation.

#### 3.2.4. Pressurized Water and Pressurized Fluid Extraction

Pressurized liquid-based technologies are also emerging as important approaches in PBP extraction. Increasing pressure as a process parameter can not only physically disrupt cell structures (e.g., high-pressure homogenization) but also enhance the solubility and mass transfer of target molecules in the solvent (e.g., pressurized liquid extraction) [82,91]. A recent study by de Sousa et al. [92] demonstrated pressurized water extraction of R-PE from the red alga *Solieria filiformis* at pressures up to 500 bar. In this work, R-PE was successfully extracted using the synergistic effect of mechanical stirring and ultrapure water, achieving a yield of 22.53 mg/g for the first time in this species. High-pressure homogenization has also been reported to significantly enhance protein extraction efficiency and can be integrated with other techniques such as EAE and UAE [88,93]. Overall, pressurized systems are highly attractive for industrial application due to their efficiency and scalability; however, their tendency to co-extract non-target compounds remains an important limitation that must be addressed in downstream purification.

#### 3.2.5. Deep Eutectic Solvents (DES)

**Figure 3:**
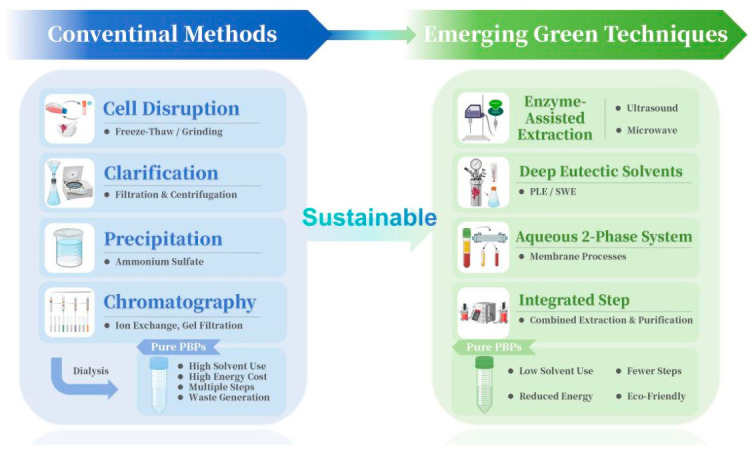
Extraction and purification workflow (conventional vs. green). Illustrative elements were created in BioRender. Suleria, H. (2026). https://BioRender.com/uix6pms (accessed on 22 May 2026).

DES and natural DES (NADES) are increasingly being explored as “next-generation solvents.” They are typically composed of hydrogen bond acceptors (HBA) and hydrogen bond donors (HBD) and can enhance the disruption of cell-wall structures and polysaccharide matrices through hydrogen-bonding networks and related interactions. At the same time, they may stabilize protein structures to some extent, while also offering green chemistry advantages such as low volatility and low toxicity [94,95,96]. A recent study reported that DES combined with UAE can significantly improve the extraction efficiency of B-phycoerythrin, achieving yields approximately 13.5 times higher than water extraction under optimized conditions. The extraction workflow can be further integrated with downstream operations such as precipitation and UF to obtain purified pigments [97]. In addition to extraction applications, DES can also be incorporated into ATPS as an alternative to conventional PEG-salt systems, thereby reducing chemical consumption and improving separation efficiency. Xu, Wang, and Hou [64] further reported a combined DES-ATPS and ammonium sulfate precipitation strategy for purifying R-PE from *Neopyropia yezoensis* (previously known as *Porphyra yezoensis*), in which the purity index (A565/A280) increased from 0.713 to 3.825. This suggests that phase-separation approaches can partially reduce reliance on multi-step chromatographic purification. An additional emerging direction is the use of NADES for single-step extraction coupled with pre-formulation, where the eutectic medium is retained in the final product. This approach can eliminate intermediate processing steps, thereby reducing time- and energy-intensive operations and minimizing process interruptions between recovery and stabilization, although an average aging-related loss of around 10% has been reported [98]. Overall, DES/NADES systems demonstrate strong potential for integrated and scalable PBP processing. The comparison between conventional purification pathways and emerging green extraction strategies for PBPs is illustrated in Figure 3.

Although Figure 3 provides an overview of the extraction and purification workflow, the practical differences among the main extraction methods are not fully shown. Therefore, Table 3 summarizes their roles, advantages, and limitations.

### 3.3. Purity Index and Standards for Food-Grade PBPs

Since PBPs are chromoproteins, the most commonly used quality control approach is the spectrophotometric purity index, defined as the ratio of absorbance at the pigment’s maximum absorption wavelength to that at 280 nm (total protein content). For PE, the A565/A280 ratio is widely adopted as a purity index, and across multiple studies, the purity criteria for R-PE are typically defined based on an absorption maximum around 565 nm [101]. However, it is also recommended to record the full UV-Vis spectrum (250–720 nm), as R-PE generally exhibits characteristic absorption peaks in the visible range at approximately 497, 538, and 565 nm [43]. Kovaleski, Kholany, Dias, Correia, Ferreira, Coutinho, and Ventura [56] reported that, for PE, purity values greater than 4.0 correspond to analytical grade, around 3.9 to reagent grade, and approximately 0.7 to food-grade protein. For PC, the A620/A280 ratio is commonly used as the standard purity index. For example, C-PC extracted from *Limnospira platensis* (formerly *Spirulina platensis*) LEB-52 was evaluated using this ratio [102]. Another review similarly defined PC purity using A620/A280 and reported grading thresholds for food, cosmetic, reagent, and analytical applications corresponding to values of >0.7, >1.5, >3.9, and >4.0, respectively [103]. For APC, A620/A280 is generally used as the purity indicator, although A_650_/A_280_ is also commonly applied as an approximation in the literature [43,104]. APC purity is often evaluated using a grading system analogous to that used for PE. In addition, regardless of the specific PBP analyzed, potential spectral interferences should be carefully considered and reported, such as the chlorophyll absorption band around ~680 nm or overlapping signals from other PBPs [104,105].

### 3.4. Challenges in Large-Scale Production

Despite continuous optimization of laboratory-scale processes, the industrial scale-up of PBPs remains primarily constrained by several key factors. First, pigment content and the extent of polysaccharide co-extraction in red algal raw materials vary seasonally, and such variability can significantly affect both extraction efficiency and purification performance. This issue is consistently highlighted as a core operational challenge in reviews on seaweed protein extraction [58,106]. Second, instability during processing and storage remains a major limitation, particularly with respect to heat, light, oxygen, and pH. Owing to the high sensitivity of PBPs, these factors can lead to degradation, yield loss, and undesirable color shifts. Consequently, environmental instability during processing and storage represents a major barrier to food applications of PBPs and has driven the development of various stabilization strategies [105].

Another major limitation is the cost and scalability of downstream processing. Although chromatographic techniques can achieve high PBP purity, they are difficult to scale up because large-volume processing requires high buffer consumption, long operation time, product dilution, and strict control of binding and elution conditions. This limitation is also related to the way PBPs are released and separated during processing. Although PBPs are water-soluble, their extraction from red algal biomass is still affected by the compact tissue structure and the polysaccharide-rich matrix, which may slow solvent penetration and protein diffusion and increase the viscosity of the extract [56,86]. In wet *Grateloupia turuturu*, ultrasound-assisted enzymatic hydrolysis improved R-PE extraction, but the yield gradually plateaued with prolonged treatment, and higher temperature reduced R-PE recovery [86]. This suggests that stronger disruption conditions do not necessarily lead to better recovery, because extraction efficiency must be balanced with pigment-protein stability.

These issues become more important during downstream purification. Co-extracted polysaccharides and non-target proteins can increase membrane fouling and reduce separation efficiency during ultrafiltration, while chromatographic purification requires careful control of pH, ionic strength, resin loading, and salt-gradient elution [56,66]. Therefore, the high purity obtained by chromatography should be considered together with buffer consumption, product dilution, processing time, and scale-up cost. Alternative systems such as DES/NADES-based ATPS may partly reduce the need for multi-step chromatography, but their performance also depends on the specific solvent and phase composition. Xu, Wang and Hou [64] reported that a choline chloride–urea/K_2_HPO_4_ system improved the purity of R-PE from *Neopyropia yezoensis* (previously known as *Pyropia yezoensis*) from 0.713 to 3.825, with a yield of 69.99%, while largely maintaining its spectral and structural characteristics. However, factors such as DES amount, protein loading, extraction time, viscosity, and system saturation still affected the separation process.

Overall, the current literature on red algal PBPs still relies mainly on endpoint indicators such as yield, purity index, recovery rate, and spectral stability. Detailed kinetic, mass-transfer, and thermodynamic modeling studies remain limited. Therefore, future work should integrate time-dependent extraction behavior, viscosity control, phase partitioning, and protein stability into process optimization. This would help connect laboratory-scale extraction and purification results with industrial process design and commercial feasibility. As a result, extraction and purification strategies must balance sustainability, cost-effectiveness, and process feasibility [82]. Engineering challenges associated with emerging green technologies further complicate industrial implementation. For instance, recent studies explicitly note that although the UAE is often considered scalable, truly large-scale applications remain limited, as cavitation distribution and energy efficiency in large-volume systems can differ substantially from laboratory conditions [99]. Finally, food compatibility, residual solvent concerns, purity requirements, and regulatory and safety considerations associated with new solvents and additives also represent important barriers. Although NADES have advanced significantly in terms of mechanistic understanding and application potential, data on toxicology, metabolic fate, and regulatory approval remain limited [100].

## 4. Colorant Functionality in Foods

### 4.1. Color Characteristics

PBPs are water-soluble chromoproteins whose strong visible-light absorption arises from open-chain tetrapyrrole chromophores (phycobilins) covalently bound to a protein scaffold. In food systems, these properties confer two main advantages. First, PBPs provide vivid coloration with strong tinting strength, meaning that only small quantities are required to achieve the desired color intensity [107]. Garcia et al. [108] reported that B-PE from *Porphyridium purpureum* required only a low coloring factor to reproduce a reference commercial pink color in dairy products, demonstrating its high coloring efficiency at low dosage. Second, PBPs exhibit good dispersibility in aqueous systems. In another study, B-PE extracted from *Porphyridium purpureum* achieved food-grade purity with an A565/A280 ratio of 2.5 [109]. When applied in gin, wine, tonic water, and isotonic beverages, the authors further confirmed its potential as a natural pink colorant for a range of drink formulations. However, because the hue and intensity of PBPs are closely linked to protein conformation, their coloring performance is inherently sensitive to processing and storage conditions. Kumar et al. [110] evaluated R-PE of different purities in extruded food systems and reported that storage stability was influenced by pigment purity, light exposure, and hydration temperature. The study also found that R-PE-containing extrudates exposed to light exhibited only slight color changes (color difference (ΔE)*ab < 5), indicating relatively limited color drift under those conditions. Importantly, the final visual appearance of a product depends not only on pigment type but also on pigment purity and the optical properties of the food matrix [111]. Therefore, in practical product development, PBP evaluation should not rely solely on spectrophotometric purity indices (Aλ/A280) but should also incorporate colorimetric parameters (e.g., ΔE*ab), as these better reflect consumer-perceived color stability and fading behavior [112].

In red seaweed sources, R-PE is typically the dominant pigment protein, providing bright red to pink hues. In addition, B-PE is also reported in certain red algae and can similarly produce stable pink to reddish coloration [108,113]. However, the development of R-PC and APC derived from red algae as mainstream food colorants remains limited, mainly due to challenges related to stability and the scalability of purification processes [32]. In contrast, the application of “natural blue” colorants in the food industry is currently dominated by C-PC, typically derived from *Spirulina*, *Arthrospira,* or *Limnospira* species [6,103].

### 4.2. Factors Affecting Stability

From a food application perspective, PBPs are generally sensitive to chemical degradation induced by heat denaturation, photo-oxidation, acidic conditions, high pressure, and heavy metal ions [114]. A useful way is to conceptualize PBPs as dual-sensitive biomolecules: they behave like pigments in their susceptibility to light and oxygen, while also exhibiting protein-like sensitivity to temperature, extreme pH, and ionic environments.

#### 4.2.1. pH Effects

In relevant food application reviews by Minic, Gligorijevic, Velickovic and Nikolic [15] and Simovic et al. [115], R-PE is often described as exhibiting a relatively broad pH stability range (approximately pH 3–10), whereas PC is generally considered more stable under a narrower, mildly acidic to neutral range (pH 4–8). PBPs are not classical pH-indicator pigments like anthocyanins; however, pH can indirectly influence their stability by altering protein conformation and the microenvironment of the chromophore. Adjali et al. [116] reported that at pH 3–4, PC exhibits a decrease in A620 and an increase in A280, which were attributed to protein precipitation and conformational changes. Similarly, another study noted that PC has poor colloidal stability under acidic conditions, leading to precipitation and consequent loss of its characteristic blue color [117]. Therefore, acidic beverage systems remain challenging matrices for blue PBP-based colorants. In contrast, R-PE, as a red pigment, is more suitable for use in mildly acidic to neutral food systems; however, its application still requires strict control of processing and storage conditions, particularly temperature and light exposure.

#### 4.2.2. Heat Effects

Thermosensitivity is one of the primary barriers limiting the incorporation of PBPs into conventional thermal processing operations such as pasteurization. For R-PE derived from red algae, recent reviews have acknowledged its relatively broad pH stability range; however, its reported thermal stability threshold generally remains below 40 °C [115,118]. For PC, temperatures above approximately 45 °C are commonly considered detrimental to stability. Adjali, Clarot, Chen, Marchioni and Boudier [116] noted in a recent review that the thermal stability of PC remains insufficient for many food-processing applications, and that current industrial approaches therefore rely on mild heat treatments or protective formulation strategies. Similarly, Velickovic, Simovic, Gligorijevic, Thureau, Obradovic, Vasovic, Sotiroudis, Zoumpanioti, Brulet, Cirkovic Velickovic, Combet, Nikolic, and Minic [32] reported that exposure of R-PC to 60 °C caused adverse and irreversible losses in both color and antioxidant activity. However, the same study also demonstrated that alginate encapsulation could significantly improve thermal stability. Interestingly, Galetovic et al. [119] subjected purified PE and PC solutions to ultra-high-temperature (UHT) treatment (138 °C for 4 s) and observed only approximately 10% denaturation for PE and 15% for PC. These findings suggest that PBPs may retain partial stability under very short-term high-temperature conditions when processing parameters are carefully controlled. Nevertheless, heat sensitivity remains a major limitation for the broader application of PBPs in conventional food processing.

#### 4.2.3. Light and Oxygen Effects

Photodegradation and oxidation often occur simultaneously in PBPs: light exposure accelerates oxidative reactions, which subsequently damage chromophores and result in pigment fading. Under aerobic conditions, the presence of additional pro-oxidant factors, such as certain metal ions, can further intensify discoloration. It has been reported that under continuous high-light exposure, the retention rate of PC decreased to only 20.2% after five days [120]. In contrast, under light-protected conditions, the retention rate remained as high as 86% over the same period, confirming the pronounced detrimental effect of light on PC stability and coloration. Rinalducci et al. [121] further demonstrated that intense visible-light irradiation of isolated phycobilisomes, which are rich in PBPs, induces the formation of ROS such as singlet oxygen (^1^O_2_) and superoxide anion radicals (O_2_^•−^). These reactive species subsequently promote oxidative damage and degradation of the protein complex. In addition, the presence of certain metal ions can further destabilize PBPs. Several studies have shown that metal ions significantly influence the properties of PBP complexes, with Cu^2+^ exhibiting particularly strong destabilizing effects [60,122]. Therefore, practical application of PBPs in food systems requires careful consideration of light-protective packaging, control of headspace oxygen, and incorporation of antioxidant protection systems. After reviewing the major factors affecting natural food colorants, Divya, Joshi, Appukuttan, Chandrapala, and Majzoobi [1] proposed stabilization strategies including the use of antioxidants, metal chelators, and protective packaging materials.

### 4.3. Role as Food Colorants

#### 4.3.1. Beverages

Beverages are considered one of the most suitable application systems for PBPs because these pigments readily disperse in aqueous media. However, beverage matrices also present some of the most challenging environmental conditions for PBP stability. Low pH, dissolved oxygen, light exposure through transparent packaging, and thermal processing can all accelerate pigment degradation and fading. For blue beverages, recent studies have primarily focused on evaluating the feasibility of C-PC as a natural blue colorant. Ghosh et al. [123] incorporated C-PC into various non-alcoholic carbonated beverages and discussed the practical importance of refrigerated storage and light-protective packaging for maintaining pigment stability. The study also reported that beverages containing preservatives and sucrose exhibited improved C-PC stability, with shelf-life retention ranging from approximately 0.5 h to 27 days depending on formulation conditions. Several studies have also explored strategies to improve pigment stability under acidic conditions. For example, Wu, Xue, Liu, Wang, Chen, Chen, Chiou, Zhou, Jiao, and Zhong [117] demonstrated that complexes formed between gelatin and PC can inhibit aggregation in acidic environments and thereby preserve color stability. In contrast to the relatively advanced commercialization pathway of C-PC, the direct industrial application of R-PE from red algae in beverages remains at an early stage. Most current research continues to focus on the physicochemical characteristics and stability of R-PE, although some studies have begun to evaluate its commercial potential. For instance, Carmona, Murillo, Lafarga, and Bermejo [109] extracted R-PE from *Porphyridium purpureum*, assessed its coloring performance in various commercial beverage systems, and discussed its applicable pH range. Overall, comprehensive reviews on natural pigments generally regard PBPs as highly promising food colorants; however, compared with well-established plant-derived pigment systems, their broader commercialization is still constrained by limitations in stability and supply-chain scalability.

#### 4.3.2. Confectionery

Color plays a critical role in confectionery products, as consumers often associate highly saturated colors with stronger flavor intensity. Consequently, PBPs possess clear visual advantages for such applications. However, the primary limitation of PBPs in the confectionery industry remains their sensitivity to processing temperatures, particularly during sugar boiling and hot-filling operations. A recent study demonstrated the feasibility of using PC as a natural blue colorant in toffee candies, emphasizing that acceptable color stability can be achieved when processing conditions are carefully controlled, specifically by adding PC after the syrup has cooled to 50–55 °C [124]. As a red pigment, R-PE could theoretically complement or compete with pigments such as anthocyanins and betanin, partly because of its comparatively broader pH stability range. Nevertheless, its sensitivity to environmental conditions still necessitates more precise process control and formulation strategies. Research on other natural pigments also provides useful insights for future PBP applications. For example, a study on betanin successfully incorporated the pigment into nanoliposomes, demonstrating that nanoencapsulation can improve both pigment stability and bioavailability [125]. These findings suggest that encapsulation-based delivery systems may offer a promising approach for enhancing the stability and functionality of PBPs in confectionery products.

#### 4.3.3. Dairy and Plant-Based Alternatives

PBPs possess inherent advantages for applications in dairy and dairy-alternative products, largely because cold-chain storage conditions can reduce thermal denaturation and pigment degradation. Multiple studies have successfully incorporated C-PC into dairy products such as milk-based ice cream and yogurt, reporting satisfactory color stability during storage periods of up to six months [126,127,128]. At the same time, several practical applications of red algal PE in dairy systems have also been reported. Garcia, Longo, Murillo, and Bermejo [108] demonstrated that B-PE can serve as a pink colorant in commercial milk-based products, achieving the desired coloration at low dosage levels while maintaining stability over an 11-day evaluation period. Pereira et al. [129] further applied PE extracted from *Gracilaria gracilis* in pancakes and yogurt formulations. In another study, both PE and PC were incorporated into yogurt and cream cheese, where they demonstrated potential not only as colorants but also as antimicrobial and antioxidant agents [130]. Furthermore, from an optical perspective, the same pigment may exhibit substantially different L* values and color intensities depending on product transparency, emulsion structure, and matrix composition [131,132]. Therefore, color calibration and formulation optimization must be performed within the intended food matrix. In future applications, the use of standardized chromaticity parameters and clearly defined measurement methodologies will also be essential for accurate evaluation and product development.

#### 4.3.4. Meat Analogues

In the field of meat analogues, color design has become a critical factor influencing consumer acceptance. In particular, meat analogue products are expected to exhibit visually recognizable color transitions during cooking that resemble those of conventional meat products [133]. In this context, available reports suggest that PE can impart pink-to-red coloration and may undergo color changes at temperatures typical of meat cooking processes [134]. This indicates that PBPs have already begun to be evaluated as candidate colorants in practical food systems. At present, direct applications of PBPs in meat analogues remain relatively limited. However, Kumar, Gaber, Knezevic, Arnesen, Moller, Ilmjarv, and Dalsgaard [110] provided important evidence supporting the feasibility of using R-PE in this sector. Their study systematically evaluated the performance of red seaweed-derived R-PE in an extruded plant-protein matrix and investigated the effects of light exposure on pigment stability. The authors also assessed R-PE retention and color characteristics in meat analogue products before and after frying. The results demonstrated that exposure to extrusion processing conditions made color retention challenging, while the final visual appearance was strongly influenced by storage lighting conditions, hydration temperature, and pigment purity. Despite these limitations, the outcomes observed after light-protected storage and frying were encouraging, supporting the future potential of PBPs in meat analogue applications. In addition, some studies have directly incorporated algal ingredients into plant-based meat systems and similarly reported notable color modifications [135]. Overall, based on their coloration properties and reported antioxidant activities, PBPs represent promising candidates for next-generation meat analogue formulations.

To summarize the current food applications of red algal PBPs, the major representative food systems, functional roles in formulations, and reported outcomes are presented in Table 4.

### 4.4. Comparison with Synthetic and Other Natural Colorants

Although increasing consumer demand for healthier and more natural products has accelerated the development of natural colorants, these pigments are unlikely to match the brightness, stability, and low cost of synthetic colorants in the near term [136]. Another reason for the continued preference for synthetic colorants in industry is their broader applicability and comparatively low sensitivity to formulation and system compatibility issues [137]. Nevertheless, the growing demand for clean-label products, increasing concerns regarding artificial additives, and the marketing value of naturally sourced ingredients are expected to continue driving the expansion of the natural colorant market. As emphasized in a systematic review of the major classes of natural colorants, including betalains, anthocyanins, carotenoids, chlorophylls, and PBPs, comparing their sources, chemical characteristics, and application limitations, in many cases, the principal limitation of natural colorants is not inadequate coloring intensity, but rather insufficient stability during processing and storage [138]. Table 5 summarizes the major classes of natural colorants, together with their key advantages, limitations, and most suitable application scenarios.

## 5. Bioactivity and Health Benefits

**Figure 4:**
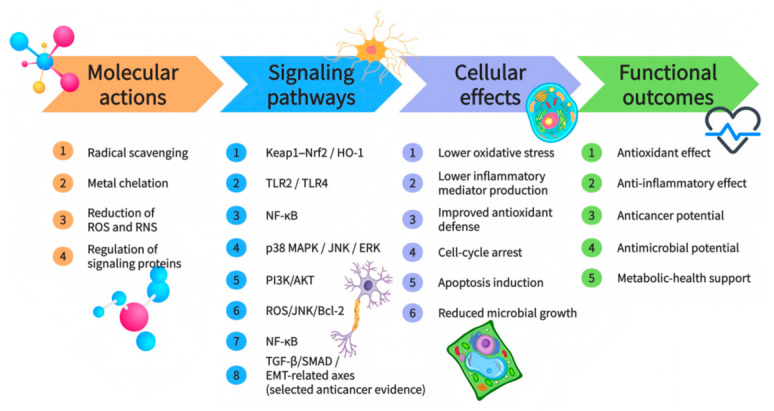
Mechanisms underlying the health-promoting effects of phycobiliproteins.

Beyond their function as natural colorants, PBPs have also been associated with a wide range of potential health-promoting effects. Figure 4 summarizes the major molecular mechanisms, signaling pathways, cellular responses, and functional outcomes reported in the literature.

### 5.1. Antioxidant Activity

In in vitro chemical systems and cellular models, PBPs, particularly C-PC derived from *Spirulina* and its chromophore PCB, have frequently been reported to possess the ability to scavenge ROS and reactive nitrogen species (RNS), thereby reducing oxidative damage markers such as lipid peroxidation [145]. Related reviews have emphasized that free-radical scavenging represents one of the principal antioxidant mechanisms of these compounds, while also citing early pioneering studies demonstrating that PCB can effectively neutralize peroxynitrite, a representative RNS [146]. Another review reported that PC can function as a non-enzymatic antioxidant by directly reducing intracellular oxidative stress through ROS scavenging [19]. In addition to direct antioxidant effects, C-PC and PCB may indirectly regulate intracellular redox homeostasis by enhancing endogenous antioxidant defense systems and suppressing pro-oxidant pathways. For example, several in vitro studies have shown that C-PC can covalently interact with Kelch-like ECH-associated protein 1 (KEAP1), thereby releasing nuclear factor erythroid 2–related factor 2 (Nrf2) and promoting the expression of antioxidant-related enzymes such as heme oxygenase-1 (HO-1). This mechanism may simultaneously modulate oxidative stress and inflammatory responses, helping to explain the frequently reported “antioxidant-anti-inflammatory coupling” associated with PBPs [147]. Meanwhile, PCB has also been reported to inhibit reduced nicotinamide adenine dinucleotide phosphate (NADPH) oxidase, a major intracellular source of ROS, while promoting the expression of antioxidant enzymes and defense systems, including glutathione-related pathways [145,148]. Beyond PC and PCB, PE has likewise demonstrated notable antioxidant potential. It has been reported that peptide fragments with molecular weights below 3 kDa, generated after simulated gastrointestinal digestion of R-PE, exhibited strong antioxidant activity in vitro [8]. Overall, however, clinical evidence for PBPs derived from red algae remains limited. Mechanistic research has been dominated largely by studies on C-PC and PCB, whereas PE has received comparatively less attention.

In terms of evidence supporting the antioxidant effects of PBPs in humans, current clinical studies primarily focus on *Spirulina* or C-PC-rich preparations rather than PBPs derived from red algae. One representative example is a randomized, double-blind, placebo-controlled trial in which patients with metabolic syndrome received 12 weeks of supplementation with *Arthrospira* liquid extract (Spirulysat^®^), a preparation rich in C-PC and polysaccharides. The study reported improvements in certain oxidative stress-related endpoints, such as a reduction in urinary isoprostanes; however, other biomarkers, including the oxidized low-density lipoprotein/low-density lipoprotein (oxLDL/LDL) ratio, showed no significant change [149]. Although these findings do not provide definitive clinical confirmation, they may be interpreted as preliminary evidence of potential biological relevance in humans. Stronger, albeit still indirect, evidence is provided by a recent meta-analysis. This synthesis of randomized controlled trials indicated that Spirulina supplementation is associated with reductions in inflammatory markers, including C-reactive protein (CRP), interleukin-6 (IL-6), and tumor necrosis factor-α (TNF-α), along with trends toward improved oxidative stress indicators such as decreased malondialdehyde (MDA) and increased total antioxidant capacity (TAC) [150]. Nevertheless, substantial heterogeneity across studies in terms of formulations, dosages, and study populations limits the strength of these conclusions. It is also important to note that, even for C-PC, authoritative reviews emphasize the lack of well-designed clinical trials specifically evaluating purified C-PC and/or PCB [145]. Accordingly, existing human evidence should be interpreted as supporting PBPs as potential functional ingredients rather than confirming clinically validated efficacy. Direct evidence from human trials using standardized PBPs derived from red algae remains largely absent. Similarly, reviews on PE highlight the need for further studies on bioavailability, dietary interactions, and toxicological safety to support its future development and application [20].

### 5.2. Anti-Inflammatory Activity

Numerous in vitro and animal studies have reported that PBPs, particularly C-PC, can exert relatively stable anti-inflammatory effects under inflammatory stimulation conditions [19]. Mechanistically, the anti-inflammatory activity of PC is primarily associated with modulation of the Toll-like receptor (TLR) and nuclear factor kappa B (NF-κB) signaling pathways. Activation of TLR2 or TLR4 initiates downstream cascades involving p38, NF-κB, and extracellular signal-regulated kinase (ERK)–activator protein 1 (AP-1) pathways signaling, ultimately leading to increased production of pro-inflammatory mediators such as TNF-α and IL-6 [151]. In contrast, C-PC is proposed to exert anti-inflammatory effects by inhibiting TLR2- and TLR4-mediated signaling. Within this inflammatory cascade, TLRs act upstream of NF-κB, a central transcriptional regulator of inflammation that controls the expression of multiple pro-inflammatory cytokines [14]. Several review studies have further summarized that C-PC can suppress NF-κB activation in disease models, resulting in decreased levels of key inflammatory cytokines, including TNF-α, IL-6, and interleukin-1 beta (IL-1β) [145]. Consistently, experimental evidence in immune cells, particularly macrophages, has shown that C-PC can inhibit NF-κB activation and reduce inflammatory mediator expression in lipopolysaccharide (LPS)-induced macrophage models [152]. In addition, because oxidative stress is closely linked to inflammatory responses, the KEAP1-Nrf2 signaling pathway, also involved in the antioxidant effects of PBPs, may represent another important regulatory mechanism underlying their anti-inflammatory activity [151].

Compared with PC, PE, including both B-PE and R-PE, still has a relatively early-stage evidence base regarding anti-inflammatory activity, with recent studies mainly focusing on intestinal inflammation and immune regulation. A study on B-PE reported multi-endpoint improvements in a dextran sulfate sodium (DSS)-induced colitis model and associated bone loss, providing a preliminary theoretical basis for its potential as an intervention molecule [153]. The study further suggested involvement of signaling pathways such as phosphoinositide 3-kinase/protein kinase B (PI3K/AKT), indicating that PE may exert protective effects through mechanisms linking inflammatory regulation and bone metabolism. In addition, other studies have investigated red algal R-PE in the context of immune signaling pathways, including the TLR4/NF-κB axis, highlighting immunomodulatory effects that are broadly consistent with those reported for PC and supporting the potential immune-regulatory activity of R-PE [154]. Overall, however, the available evidence remains largely preclinical. Given that several mechanistic pathways have not yet been fully validated, a substantial gap remains before firm conclusions can be drawn regarding clinical translation.

### 5.3. Anticancer Potential

Existing research on the antitumor potential of PBPs (including PE, C-PC, and APC) remains largely limited to in vitro cell-based models. Multiple reviews have reported that PBPs may inhibit cancer cell proliferation and metastasis, disrupt cell cycle progression, and induce apoptosis [19,155,156]. With respect to R-PE, Tan et al. [157] demonstrated that it can inhibit the proliferation of SGC-7901 gastric cancer cells and induce apoptosis by interfering with S-phase cell cycle progression. Mechanistically, R-PE was shown to downregulate cell division cycle 25A (CDC25A) expression, thereby inhibiting cyclin-dependent kinase 2 (CDK2) activation and reducing Cyclin-CDK complex activity, ultimately resulting in synthesis-phase cell cycle arrest. More recent studies have further expanded this evidence base. PE derived from *Gracilariopsis lemaneiformis* significantly inhibited the proliferation of SKOV-3 ovarian cancer cells and induced apoptosis via modulation of the reactive oxygen species (ROS)/c-Jun N-terminal kinase (JNK)/B-cell lymphoma 2 (Bcl-2) signaling axis [158]. In addition, PE extracted from *Kappaphycus alvarezii* has been reported to reduce cell viability and promote apoptosis in human lung cancer cells, further supporting the potential anticancer bioactivity of red seaweed-derived PE beyond its role as a natural colorant [47].

Evidence from non-red algal PBP sources further supports the broader anticancer potential of phycoerythrin. Zhao et al. [159] reported that PE isolated from *Rhodomonas salina* can induce apoptosis in human lung cancer A549 cells, suggesting that its mechanism may involve signaling pathways such as ERK/Bcl-2 homologous antagonist/killer (Bak) and JNK/caspase-3. In the case of C-PC, a study demonstrated that it can induce apoptosis in breast cancer MDA-MB-231 cells through activation of p38 mitogen-activated protein kinase (p38 MAPK) and JNK pathways, while simultaneously inhibiting the ERK signaling pathway [160]. In addition, the classical NF-κB pathway has also been implicated in its anticancer effects. For example, Hao et al. [161] reported that C-PC can modulate NF-κB signaling, thereby influencing the phenotypic behavior of non-small cell lung cancer cells. Furthermore, C-PC has been investigated in combination with therapeutic strategies. For instance, [162] reported its potential as a radiosensitizer, where it enhanced the efficacy of radiotherapy in colorectal cancer by inhibiting cyclooxygenase-2 (COX-2) expression.

In contrast, direct in vivo evidence for the antitumor effects of PBPs remains limited and highly heterogeneous. Pan et al. [163] reported the antitumor activity of R-PE in both an in vitro HeLa cell model and an in vivo sarcoma 180 (S180) tumor-bearing mouse model. Huang et al. [164] employed BALB/c nude mice to establish tumor metastasis and xenograft models and linked the observed effects to modulation of the Akt/β-catenin pathway, epithelial–mesenchymal transition (EMT), and related signaling mechanisms. Similarly, Chen et al. [165] investigated both in vivo (HEC-1A cell implantation in female nude mice) and in vitro (HEC-1A and Ishikawa cell lines) models, demonstrating that APC and C-PC can suppress endometrial cancer metastasis by targeting the transforming growth factor-beta (TGF-β)/SMAD signaling pathway and reversing EMT. However, it is important to emphasize that these studies differ substantially in terms of protein purity, structural state, dosage, and exposure duration. As a result, the overall evidence remains restricted to the preclinical level. Even within the same PBP category, the biological activities of high-purity APC and C-PC may vary significantly [166].

### 5.4. Antimicrobial Properties

Garcia-Gomez, Aguirre-Cavazos, Chavez-Montes, Ballesteros-Torres, Orozco-Flores, Reyna-Martinez, Torres-Hernandez, Gonzalez-Meza, Castillo-Hernandez, Gloria-Garza, Kacaniova, Ireneusz-Kluz and Elizondo-Luevano [14] noted that PBPs and their chromophores (phycobilins) exhibit exploratory potential in antibacterial, antifungal, and antiviral applications, and summarized the relevant reported activities. However, existing studies predominantly focus on cyanobacterial PBPs rather than compounds derived from red seaweeds. In terms of antibacterial and antifungal effects, most current research remains at the level of in vitro screening or crude extract evaluation. For example, C-PC extracted from *Jaaginema minimum* (formerly *Oscillatoria minima*) (Cyanobacteriophyta) has been reported to inhibit a range of bacterial strains [167]. In addition, Nowruzi et al. [168] incorporated cyanobacterial PE (from *Nostoc* sp. Ft salt) into fresh rainbow trout filets and observed a significant reduction in microbial growth compared with untreated controls. Notably, Balasundaram, Seethapathy, Sankaralingam, Mahendran, Mareeswaran, Pandiarajan, Maheswari, Venkatesh, Arunkumar, Pathade, Pandita, Pandita, Ullah, Elansary, Nazim, Fickak, Rashwan and Moussa [47] demonstrated that PE isolated from the red alga *Kappaphycus alvarezii* (Doty) exhibited antibacterial activity against *Klebsiella oxytoca* and *Proteus mirabilis*. Regarding antifungal activity, PBPs extracted from *Limnospira platensis* (Cyanobacteriophyta) and *Gracilaria cornea* (formerly *Hydropuntia cornea*) have been shown to significantly inhibit mycelial growth and spore germination of Botrytis *cinerea* [169]. Although inhibitory effects have been reported, the literature is highly heterogeneous in terms of raw material sources, extraction and purification methods, and bioactivity assessment systems. These inconsistencies substantially limit cross-study comparability, and therefore conclusions should be interpreted with appropriate caution.

In terms of antiviral activity, Pendyala et al. [170] reported that PCB can inhibit both the main protease and papain-like protease of coronaviruses (CoVs), suggesting its potential as a phytochemical antiviral inhibitor. More recently, Garcia-Gomez, Aguirre-Cavazos, Chavez-Montes, Ballesteros-Torres, Orozco-Flores, Reyna-Martinez, Torres-Hernandez, Gonzalez-Meza, Castillo-Hernandez, Gloria-Garza, Kacaniova, Ireneusz-Kluz and Elizondo-Luevano [14] summarized multiple lines of evidence supporting the antiviral potential of PBPs, including the activity of C-PC against influenza A (H1N1) virus in in vivo models and the inhibitory effects of APC on the replication of enterovirus-71 [14]. From a food and health claims perspective, however, the current evidence supporting antibacterial and antiviral activities remains insufficient for substantiating functional health claims. At this stage, such effects are more appropriately regarded as exploratory research directions rather than validated functional outcomes. Nevertheless, the antiviral potential of PBPs and their chromophores remains an important area for further investigation.

### 5.5. Metabolic Health

Evidence related to C-PC is most extensively represented in the field of metabolic health; however, the current evidence base remains primarily grounded in mechanistic and preclinical studies. Importantly, the *Spirulina* system currently serves as the most relevant human evidence benchmark for assessing the translational potential and evidentiary limitations of red seaweed-derived PBPs. Ziyaei et al. [171] conducted a comprehensive database search and reported that, as of 30 April 2023, the available evidence was derived predominantly from cell-based and animal studies, clearly highlighting a significant gap in clinical validation.

The latest Grading of Recommendations Assessment, Development and Evaluation (GRADE)-assessed dose–response meta-analysis of randomized controlled trials indicates that *spirulina* supplementation can produce small but statistically significant improvements in anthropometric outcomes, including body weight (weighted mean difference (WMD): −1.07 kg), body mass index (WMD: −0.40 kg/m^2^), and body fat percentage (WMD: −0.84%), suggesting that higher doses and longer intervention durations may yield more pronounced effects [172]. For glycemic outcomes, a meta-analysis of 35 randomized controlled trials (RCTs) further reported significant improvements in fasting blood glucose (FBG) (−5.51 mg/dL), insulin resistance [homeostatic model assessment of insulin resistance (HOMA-IR): −0.68], and insulin levels (−0.86 ng/mL) [173]. With regard to lipid profiles, reductions in triglycerides, total cholesterol, and low-density lipoprotein cholesterol (LDL-C), along with increases in high-density lipoprotein cholesterol (HDL-C), were also observed; for instance, LDL-C decreased by a weighted mean difference of −7.69 mg/dL in their analysis. Overall, existing clinical meta-analyses suggest that *spirulina* products may improve multiple metabolic health indicators; however, the certainty of evidence varies across outcomes, with some endpoints rated as low certainty under GRADE. In addition, most trials have used whole *spirulina* biomass or composite extracts, making it difficult to attribute observed effects specifically to individual components such as C-PC. Consistently, [149] reported that Spirulysat^®^ (a *spirulina* aqueous extract rich in C-PC) reduced urinary isoprostanes, decreased triglycerides, and increased HDL-C in individuals with metabolic syndrome, although it had no significant effect on the oxLDL/LDL ratio. For red seaweed-derived PBPs (such as R-PE or APC), it is therefore more appropriate at present to consider metabolic health applications as a potential research direction rather than an established clinical outcome. There remains an urgent need for well-designed human RCTs and meta-analyses based on standardized formulations and dosing regimens to establish an evidence base comparable to that of *spirulina*-derived systems.

Table 6 provides a summary of the reported biological activities of red seaweed-derived PBPs and the supporting evidence.

### 5.6. Synergistic Dual Roles in Foods

The development goals of PBPs (PE, PC, and APC) from red algae in food applications are often multifaceted: a single ingredient is expected to provide both consumer-perceivable coloration and measurable health benefits. Accordingly, PBPs are frequently discussed in the literature as dual-function ingredients that combine natural food colorant properties with bioactive potential, which further highlights their multifunctional role in food systems [182]. However, their practical implementation in real food matrices is constrained by high sensitivity to heat, light, and acidic conditions, which can result in insufficient stability, reduced bioavailability, and, in some cases, undesirable off-flavor formation [116]. Therefore, the use of carrier systems and structure-guided formulation strategies is often necessary to enhance stability and enable controlled functional delivery of PBPs [105,114].

## 6. Stability of PBPs in Food Matrices

### 6.1. Protein Network

In dairy and plant-protein gel systems (e.g., yogurt and soy protein gels), color expression depends not only on the intrinsic light absorption of pigments but also on the optical properties of the matrix, particularly light scattering. Several studies have shown that protein networks and dispersed fat droplets can significantly increase the scattering coefficient, thereby altering perceived color intensity and lightness [183,184]. Consequently, identical pigment concentrations may yield markedly different L* values and visual color strength across formulations.

At the same time, interactions between PBPs and food proteins can create microenvironmental effects that influence pigment stability. For example, Zhang et al. [185] reported that PC can interact with whey protein isolate (WPI), improving its stability under acidic and light-exposed conditions. Similarly, [186] demonstrated that native whey proteins can enhance the colloidal stability of PC in acidic systems, suggesting a potential protective role of protein-based matrices.

However, this protective effect is highly condition-dependent and may be reversed under stress conditions. Under acidic pH combined with thermal or oxidative stress, PC can undergo structural destabilization, leading to pronounced spectral changes indicative of chromophore disruption and protein unfolding [116]. In addition, phase instability may occur in protein-rich systems, where PBPs co-precipitate with whey proteins under unfavorable pH conditions, resulting in pigment loss from the dispersed phase. Such co-precipitation is often accompanied by increased turbidity and visible sediment formation, ultimately leading to significant deterioration in color quality [187].

### 6.2. Polysaccharide Systems

In polysaccharide-rich food matrices (e.g., pectin and carrageenan systems), the stability of PBPs is largely governed by the formation of soluble and colloidally stable protein-polysaccharide complexes under acidic and thermal processing conditions. In general, anionic polysaccharides can enhance the stability of PC under low-pH environments by promoting electrostatic interactions that suppress protein aggregation and precipitation, thereby improving both colloidal stability and color retention after heat treatment [188].

However, protein-polysaccharide interactions are highly sensitive to environmental conditions, particularly pH and ionic strength. Rodríguez Patino and Pilosof [189] noted that inappropriate conditions can induce associative phase separation, leading to the formation of insoluble complexes and subsequent precipitation. They further emphasized that the protein-to-polysaccharide ratio is a critical determinant of system behavior: insufficient polysaccharide coverage may promote bridging flocculation, driving the system from a stable dispersion toward aggregation and sedimentation. [189].

Among different polysaccharide systems, carrageenan has been most extensively studied for PBP stabilization. A systematic investigation demonstrated that λ-carrageenan can significantly improve the thermal and color stability of PC under acidic conditions (pH 2.5–6.0) and elevated temperatures (70–90 °C), primarily through complex formation that protects the pigment structure [190]. In addition, molecular weight was shown to be a key factor Buecker et al. [191], with higher molecular weight λ-carrageenan exhibiting stronger binding capacity and superior color retention during heating and storage.

Pectin-based systems are particularly relevant for beverages and acidic formulations. A study on pectin-PC complexes in model beverage systems showed that complexation can effectively reduce color degradation during storage and improve particle size stability under combined thermal processing and storage conditions [192].

### 6.3. Fat Phase and Emulsion Structure

In food matrices containing a lipid phase (e.g., dairy beverages, desserts, sauces, and plant-based milk), PBPs should be considered not only as colorants but also as potential functional structuring agents. As protein-based macromolecules, PBPs exhibit inherent amphiphilicity and interfacial activity, enabling partial adsorption at the oil-water interface. This interfacial adsorption can contribute to the formation of protein-stabilized films, thereby influencing droplet organization, light scattering behavior, and overall emulsion stability [193]. Galani et al. [194] demonstrated that C-PC, when combined with pea protein isolate, can act synergistically in oil-in-water emulsions. The study showed that PBPs contribute to emulsification performance by reducing interfacial tension, modifying droplet size distribution, and improving storage stability, highlighting their dual role as both functional pigments and interfacial stabilizers.

Moreover, structural modification and composite assembly with polysaccharides or polyphenols can further enhance the interfacial functionality of PBPs. For instance, Gong et al. [195] developed oil-in-water emulsions containing thyme essential oil and coconut oil stabilized by plasma-modified PC (50 W, 20 s). The resulting system exhibited a more compact interfacial structure, improved microstructural integrity, and antimicrobial activity against *E. coli*. Similarly, Hao et al. [196] fabricated a Pickering emulgel using PC nanoparticles and cationic starch. The resulting system showed excellent stability against heat treatment, pH variation, and freeze–thaw cycles, along with enhanced UV-shielding properties. Collectively, these findings suggest that PBPs can extend beyond their traditional role as pigments and function as multifunctional interfacial building blocks in complex food colloidal systems.

### 6.4. Critical Strategies

#### 6.4.1. Microencapsulation

Spray drying and microencapsulation are important industry-friendly approaches for the industrialization of PBPs. They can protect PBPs by entrapping the pigment-protein complex within wall materials, thereby forming a barrier that prevents or reduces direct contact with heat, light, oxygen, moisture, and unfavorable pH conditions during processing and storage [197]. By limiting these environmental triggers, the wall-material matrix may slow chromophore degradation, protein denaturation, and disruption of the native pigment-protein assembly [198]. Wall materials such as maltodextrin and gum Arabic can further improve powder stability by reducing moisture content and water activity, which helps limit moisture-related deterioration during storage [199]. One study reported that encapsulation of C-PC in chitosan microparticles (with an encapsulation efficiency exceeding 36%) effectively maintained its stability under acidic (pH 2) and alkaline (pH 10) conditions, as well as during both light and dark storage and under heat stress [200]. Vieira, Noore, Tiwari, O’Donnell, Goncalves, Pastrana and Fucinos [105] successfully employed nano spray drying, using inulin and gum arabic as encapsulating materials to enhance the stability and functionality of PBPs (PCs and PEs), including color retention and antioxidant performance.

#### 6.4.2. Nanocarriers and Composite Carriers

If the focus is on health benefits rather than shelf-life color stability alone, it becomes necessary to consider retention and release during the digestion process. Nanocarriers and composite carriers can protect PBPs by improving the physical stability of the loaded system and delaying premature release under storage- or digestion-related conditions. In chitosan-coated PC-loaded nanoliposomes, chitosan coating improved the stability of particle size, zeta potential, and polydispersity index and reduced the release of loaded PC [201]. Another study used PC-loaded nanoliposomes (NLs) as a model system and systematically compared the effects of nanochitosome (NC) and double-layered nanoliposome (NP) coatings on system performance [202]. The results showed that, compared with NLs, which retained only about 58% of PC after light exposure, NCs and NPs increased retention to approximately 69% and 77%, respectively. Meanwhile, the coated systems exhibited more pronounced controlled-release behavior in simulated gastric fluid (SGF), with cumulative release over 2 h decreasing from 67.2% (NLs) to 47.7% (NPs). Composite carriers may also be formed through protein-polysaccharide interactions. In a PC complex formed with sodium alginate and lysozyme, PC showed improved stability during exposure to light, temperature changes, different pH conditions, and simulated gastrointestinal fluids, indicating that this type of biopolymer complex can help reduce environmental and digestion-related instability [203].

#### 6.4.3. Gel and Hydrogel

In certain desserts, gel candies, 3D-printed foods, and structured plant protein products, gel systems can effectively reduce diffusion and oxygen exposure while providing color uniformity. For PBPs, gel and hydrogel systems can immobilize the pigment-protein complex within a hydrated three-dimensional network, which helps restrict pigment diffusion and supports controlled release under different pH conditions [204]. One study showed that ultraviolet B (UVB) irradiation promoted interactions between PC and alginic acid, potassium salts, and sodium salts in Undaria pinnatifida (UP), leading to the formation of a reinforced gel network [205]. The formation of hydrogen bonds not only improves the texture of the hydrogel but also enhances its thermal stability, thereby broadening its potential applications in related fields.

### 6.5. Case Study—PBPs in Real Dairy Protein Networks (Color + Antioxidant + Antibacterial)

The study by Ahmadi, Anvar, Nowruzi, and Golestan [130] provides a representative successful example. The investigated systems included low-fat yogurt (a fermented protein gel network) and cream cheese (a curd- and fat-based structured protein network), both of which are typical protein-dominated semi-solid food matrices. PBPs, including PE, PC, and their combinations, were incorporated into these real dairy protein networks, and a comprehensive evaluation of color, antioxidant activity, microbiological stability, and sensory attributes was conducted under refrigerated storage at 4–8 °C. The results showed that the addition of PBPs significantly altered the Commission Internationale de l’Éclairage L*a*b* (CIELAB) parameters: PE and PC, respectively, increased the a* and b* values of the samples, while the ΔE values of the fortified samples were lower than those of the control, indicating reduced color variation during storage. In terms of functionality, by the end of storage, the ferric reducing antioxidant power (FRAP) of PE-fortified cream cheese was approximately 2.34–2.75 times higher than that of the control, whereas PE-fortified yogurt exhibited approximately 1.71–1.80 times higher activity after 28 days. Regarding antimicrobial properties, the counts of psychrotrophic bacteria in cream cheese containing PE or the PE-PC combination decreased to approximately 0.71–0.80 times that of the control at the end of storage. In yogurt, the corresponding values were approximately 0.83–1.27 times that of the control, while mold and yeast counts in the combined treatment group also remained within acceptable standard limits. Notably, the authors emphasized that incorporating PBPs into protein networks is not merely a matter of “adding color without trade-offs”; rather, it requires careful control to mitigate potential risks such as protein hydrolysis and off-flavor development during storage.

## 7. Safety, Regulation, and Consumer Acceptance

### 7.1. Safety

#### 7.1.1. Identity and Specifications

From a regulatory perspective, establishing clear identity and specifications is the first hurdle to commercialization. For PBP-based ingredients, specifications should clearly define at least the following: source species, extraction process, indicators of the target protein or chromophore (e.g., purity and composition), limits for critical impurities (e.g., salts, residual solvents, heavy metals, and microbial contaminants), and batch-to-batch consistency. Taking the Joint FAO/WHO Expert Committee on Food Additives (JECFA) monograph on *Spirulina* extract (International Numbering System for Food Additives, INS 134) as an example, it explicitly defines the source organism and the boundaries of the aqueous extraction process, and states that the primary coloring constituents are C-PC and APC [206]. The document also specifies color value ranges, heavy metal limits (As, Cd, Pb, and Hg), and microbiological standards (e.g., total plate count, yeasts and molds, and *E. coli* indicators). In addition, it highlights the need for more comprehensive compositional characterization, identity testing, and validated analytical methods for purity assessment in future evaluations.

#### 7.1.2. Toxicology and Exposure

For PBPs intended for use as colorants, regulatory frameworks typically require dietary exposure assessments based on the intended food categories and typical or maximum use levels. These estimates are then evaluated alongside toxicological evidence for risk characterization. In the summary report of the 86th JECFA meeting, the Committee conducted a conservative exposure assessment for *spirulina* extract used as a food colorant using the budget method (expressed as PCs, approximately 190 mg/kg body weight for adults and 650 mg/kg body weight for children), concluding that this level of exposure does not pose a health concern [207]. In the same report, the Committee assigned a temporary acceptable daily intake (ADI) of “not specified” for *spirulina* extract. However, this does not indicate insufficient toxicological evidence or an identified health risk; rather, it reflects the preliminary nature of the product specifications, with a final conclusion dependent on further refinement and confirmation of these specifications. Therefore, risk characterization depends not only on toxicological and exposure data but also fundamentally on whether specifications can reliably define the identity and quality boundaries of the assessed substance, thereby ensuring consistency between batches and marketed products.

#### 7.1.3. Allergenicity

PBPs are edible proteins; therefore, when discussing safety, allergenicity should be treated as a verifiable risk factor, particularly when highly purified PBPs are incorporated into beverages, dairy products, or plant-based formulations where intake levels may be comparatively high. International practice typically follows a structured evidence pathway: it begins with in silico sequence homology screening, is followed by in vitro digestion assays (e.g., SGF or pepsin resistance testing), and is sometimes supplemented with IgE-binding assays or serological evidence to evaluate potential cross-reactivity risks [208,209]. From the perspective of regulatory labeling frameworks, PBPs derived from red seaweed are generally not classified as priority allergens under Australia–New Zealand and EU requirements [210,211]. However, sporadic clinical evidence suggests that edible seaweeds may still trigger IgE-mediated reactions. For example, clinical reports have documented IgE-mediated hypersensitivity associated with spirulina, while reviews and proteomic homology analyses further indicate that PC and other proteins may contribute to sensitization and cross-reactivity [212,213]. Although overall clinical evidence for red-algal allergy remains limited, IgE-mediated case reports involving red seaweeds such as nori, including positive skin prick test (SPT) results, warrant consideration [213,214]. Therefore, in applications involving higher-purity PBPs and increased dietary exposure, it is necessary to define risk boundaries based on the aforementioned evidence chain and to clearly articulate corresponding risk management considerations to address regulatory expectations.

### 7.2. Regulatory Landscape

#### 7.2.1. Food and Drug Administration (FDA)

In the United States, color additive regulation is not determined primarily by whether a substance is natural or plant-derived, but by its intended use for coloring purposes. When used as a color additive, a substance falls under the FDA’s color additive framework, meaning it must be explicitly listed in the regulatory inventory and is subject to specified conditions of use and regulatory limitations [215]. Spirulina extract is listed in 21 CFR 73.530, which defines permitted food categories, good manufacturing practice (GMP) requirements, and labeling obligations [216]. The FDA has also expanded its permitted food categories through multiple Federal Register rulemakings. In particular, the 2022 final rule extended its use to beverages, sauces, salad dressings, non-dairy alternatives, and other food products, with subsequent notices confirming its effective regulatory status [217]. Although this regulation does not directly apply to red seaweed-derived PBPs, it nevertheless provides an important regulatory reference point for evaluating their potential approval pathways and use conditions.

#### 7.2.2. European Food Safety Authority (EFSA)

Under the EU framework, compliance for color-related ingredients primarily depends on whether a material is classified as a food additive or a coloring food. This distinction is not determined by the algal source itself, but mainly by whether the extraction and processing steps result in selective enrichment of pigments and whether the product retains the essential characteristics of the source material [218]. It can also be assessed using indicators such as the enrichment factor, as well as the decision tree (Annex I) and checklist (Annex II) provided in relevant guidance documents [219]. These annexes refer to the annexes of the guidance document. Industry-level operational guidance further translates these requirements for ingredient definition into actionable manufacturing and labeling practices [220].

If the substance is classified as a food additive, it must be included in the EU list of authorized additives and used only within specified food categories and conditions of use. This pathway typically involves the European Food Safety Authority’s (EFSA) safety evaluation and dietary exposure assessment framework and requires labeling with the additive name or its corresponding E-number [221]. By contrast, if it is classified as a coloring food, it is generally declared as a food ingredient on the label rather than being subject to the food additive authorization system and E-number designation [219].

#### 7.2.3. Food Standards Australia New Zealand (FSANZ)

Under the FSANZ framework, the use of food additives operates under a combined system of pre-market approval and category-based restrictions. Standard 1.3.1 specifies that a substance may be used as a food additive only if it is explicitly permitted and complies with the relevant conditions of use [222]. The list of permissions and restrictions is primarily set out in Schedule 15. This schedule is structured around food-category tables in which permissions are organized under hierarchical headings with inherited authorisations, while conditions of use are defined through either maximum permitted levels (MPLs) or GMP. Ultimately, it translates regulatory permissions and limits into enforceable requirements through fields such as INS number, substance description, permitted levels, and conditions of use [223].

In the latest effective version of Schedule 15, no general permission entry is explicitly listed for PBPs such as PC or phycoerythrin. Therefore, red seaweed-derived PBPs must follow a clearly defined regulatory pathway either as food additives under the relevant permission and labeling framework or be assessed separately as novel foods. For example, Application A1263 (*Rhodomonas salina* extract as a novel food) was initially progressed but later withdrawn, highlighting both the inherently pre-market nature of this assessment pathway and the uncertainty associated with achieving market authorization [224].

### 7.3. Consumer Perceptions of Red Seaweed-Derived Ingredients

In a typical Western dietary context, seaweed is often perceived as a healthy and sustainable novel food resource; however, consumer acceptance and potential sensory concerns still significantly limit its routine consumption. A nationwide consumer survey in Australia reported that approximately 75% of respondents had tried seaweed, but only 37% could be classified as regular consumers within the past 12 months [225]. Similarly, research by Young et al. [226] indicates that among young Australian consumers, nutritional and health attributes are key drivers of purchase intention. However, the authors also note that issues such as availability and price remain major barriers to wider adoption of seaweed-based products. In New Zealand, willingness to try red seaweed appears to be more strongly influenced by individual-level factors. Structural equation modeling results suggest that consumers unwilling to try red seaweed are more likely to exhibit food neophobia, whereas greater awareness of the nutritional value of algae is positively associated with willingness to try red seaweed [227]. A cross-national study by Chen et al. [228] further indicates that different product formats can elicit varying emotional responses and acceptance levels, and sensory attributes such as sticky texture and fishy flavor may significantly reduce consumer preference.

Interestingly, consumer acceptance of seaweed-based foods is not determined solely by actual taste or flavor. Instead, informational framing can reshape expectations and influence the interpretation of sensory experiences. An experimental study by Moss et al. [229], using a seaweed smoothie as a model product, showed that when participants received either nutrition (health benefit) information or consumption-context information, hedonic liking scores increased and sensory evaluations improved, even for the same formulation. In contrast, the authors also noted that providing sustainability-related information alone appeared to increase perceptions of “fishiness” and reduce perceived sweetness. Therefore, promotional strategies for red seaweed PBP ingredients should prioritize clear communication of functional attributes (e.g., color performance and stability strategies) and safety-related information (e.g., specifications and usage limits), while also providing practical consumption contexts to reduce uncertainty and perceived risk. This interpretation is consistent with findings from an Australian study, which suggest that labels and promotional materials should guide consumers on how to purchase, store, prepare, and use seaweed products.

The connection between consumers and “clean label” perceptions is also worth noting. “Clean label” does not have a standardized regulatory definition; however, academic research and reviews generally suggest that consumers associate it with fewer processing steps, shorter ingredient lists, and more “natural” products, and they often hold negative perceptions of E-numbers or highly processed-sounding ingredients [230,231]. This implies that if red seaweed PBPs enter the market via a color additive regulatory pathway and are declared on labels using unfamiliar additive names or E-numbers, they may be associated with negative consumer perceptions and reduced acceptance.

## 8. Sustainability and Future Directions

### 8.1. Sustainable Cultivation of Red Seaweeds for PBPs

For the large-scale application of red seaweed PBPs (especially R-PE and APC), extraction technology is generally not the primary constraint. The key challenge lies in whether raw materials can be maintained in a stable, traceable, and sustainable supply system. Compared with wild harvesting, aquaculture allows better control over species origin, contaminant profiles, and batch-to-batch consistency; however, sustainability is not automatically guaranteed [232]. Site selection and environmental carrying capacity, biosecurity and disease management, environmental monitoring, as well as community engagement and governance considerations, can all directly affect the social license for industry expansion and its long-term stability [233].

For PBPs, a more critical trend is the integration of cultivation processes with quality design. By regulating light, nitrogen availability, temperature, salinity conditions, and harvest timing, the accumulation of PBPs and the relative proportions of PE, PC, and APC can be more precisely controlled. Existing evidence indicates that environmental factors can significantly influence both the total content and compositional ratios of PBPs. For example, in the economically important red seaweed *Kappaphycus alvarezii*, increased light intensity has been reported to significantly reduce PE content, whereas in *Porphyridium purpureum*, lower salinity and semi-continuous cultivation can significantly enhance PE yield while altering the overall PBP composition [12,26]. This is highly relevant in practice, as fluctuations in raw material composition directly affect downstream extraction and separation processes. The high viscosity and strong ionic interactions associated with red seaweed cell walls and intracellular polysaccharides can substantially increase protein release complexity and subsequent separation difficulty, thereby amplifying the burden on filtration, clarification, and purification steps [59]. A more feasible model is therefore the development of a “PBP-ready” supply chain, in which cultivation outputs are delivered with defined pigment-performance metrics and full traceability information [232].

### 8.2. Integration into Biorefinery Models

Red seaweeds are naturally well suited to a biorefinery concept, as their commercial exploitation is primarily based on hydrocolloids, while they also contain multiple valuable co-products such as PBPs, sulfated polysaccharides, phenolics, mycosporine-like amino acids (MAAs), and minerals, thereby providing a strong foundation for integrated multi-product valorisation [234,235]. If PBPs can be selectively recovered under mild processing conditions, they could serve as a high-value core industrial stream, potentially enhancing profitability across the entire value chain. However, this strategy is only feasible when PBPs are incorporated into a multi-product biorefinery framework, rather than developed as an isolated high-purity pigment protein industry [235,236].

Recent studies on red seaweed extracts have identified key directions for integrating extraction processes. By designing a rational extraction sequence and operating conditions, for example, employing milder steps such as cold-water or ethanol extraction to recover valuable by-products, it is possible in some cases to recover co-products while maintaining or even improving key functional properties of downstream agar and agarose polysaccharides, such as gel strength and melting point [237]. Looking ahead, green solvents and process intensification approaches (e.g., DES and NADES) may further improve separation efficiency; however, these must be critically evaluated from a food-grade perspective [238]. It should also be noted that factors such as solvent recyclability, residual solvent specifications, and regulatory acceptance ultimately determine whether such approaches can be implemented within the food supply chain [100].

### 8.3. Scale-Up and Commercialization Challenges

Although progress at the laboratory scale has been rapid, industrial-scale application remains constrained by multiple persistent challenges. In particular, downstream processing costs and limited production capacity significantly hinder scale-up. Several reviews have summarized the industrialization challenges of PBPs as a balancing issue between cell disruption, extraction, purification, and the stability requirements of end-use applications, and have emphasized the need for more cost-effective downstream processes [56,239]. Kovaleski, Kholany, Dias, Correia, Ferreira, Coutinho and Ventura [56] further noted that, in existing processing routes, purification often relies on multi-step separation strategies targeting increasingly stringent pigment-protein purity grades (from food-grade to analytical-grade), which inherently increases costs and exacerbates throughput bottlenecks. This observation is consistent with the broader consensus in the protein purification field that conventional chromatography is “high-cost and limits productivity” [240].

The limited stability of PBPs under industrial processing and storage conditions also remains an unresolved challenge. Reviews on food applications consistently emphasize that PBPs are sensitive to heat, light, oxygen, acidity, and metal ions, and that stabilization strategies, including formulation systems, carrier systems, encapsulation technologies, and protective packaging against light and oxygen, are essentially prerequisites for their incorporation into mainstream food products [114]. The sensitivity of PBPs has been discussed in detail in previous sections and is therefore not reiterated here.

Companies pursuing industrial-scale PBP production also require predictable color performance (e.g., CIELAB parameters and ΔE), clearly defined purity indices (e.g., A620/A280 for PC and A565/A280 for phycoerythrin), and well-specified impurity limits (e.g., microbiological criteria, heavy metals, and process-related residues) [103]. Regulatory authorities also establish specification requirements for key impurities. For example, U.S. regulations for *spirulina* extract (in which PC is the principal coloring component) specify limits for Pb, As, and Hg, and require non-detectable levels of microcystins [216]. Red seaweed-derived materials are significantly influenced by seasonality, harvesting location, and environmental variability. Annual monitoring studies have reported that components such as PBPs and carrageenan exhibit seasonal and spatial variation and are correlated with factors such as temperature, rainfall, and solar radiation, making batch-to-batch consistency more difficult to control [48]. Therefore, it is necessary to comprehensively consider product specifications and ensure robust batch-to-batch consistency in industrial production.

### 8.4. Emerging Applications

#### 8.4.1. Three-Dimensional Food Printing

In 3D-printed foods, color is increasingly being treated as a design variable alongside structure and nutritional composition. Recent reviews have summarized how printable colorants must balance printability, stability, and safety, and have identified natural colorants as an important component of next-generation printable formulations [241,242]. For PBPs, experimental studies have incorporated G-PC into edible films or packaging-film matrices and reported measurable changes in color parameters, alongside functional effects such as enhanced antioxidant activity and improved light-barrier properties [243,244]. These findings suggest that, provided the matrix system is appropriately matched to PBP stability requirements, it is technically feasible to achieve customizable coloration with additional functional benefits.

#### 8.4.2. Functional Beverages

Beverages represent a promising application area for PBPs. As water-soluble pigment proteins, their key advantage lies in their ability to produce bright red coloration in aqueous systems, and pigments derived from red algae are frequently described in the literature as natural ingredients with dual functionality, encompassing both coloring and bioactivity [15,107]. However, beverages also represent a high-risk application context. Reviews consistently indicate that PBPs are difficult to apply directly in acidic beverages due to their limited stability, while the stability of PE in particular depends on protection from light exposure, low temperatures, and appropriate pH conditions [8,15]. Therefore, improving stability through formulation strategies (e.g., blending and encapsulation), in combination with packaging approaches that limit light and oxygen exposure, is widely regarded as an integrated and rational solution [114,144].

#### 8.4.3. Nutritional Supplements

Compared with mainstream food products, supplements are generally easier to control in terms of dosage and standardization and are, therefore, often considered a more immediate pathway to commercialization. A review systematically discussed the bioactivities associated with PBPs and summarized examples of commercial supplements, indicating that a nutraceutical market for phycobiliprotein-related ingredients already exists to some extent, although these products are not necessarily derived from red seaweed sources [14]. Similarly, sulfated polysaccharides produced by the red microalga *Porphyridium* spp. have been explicitly reported as being used in nutraceutical products with antioxidant activity [245]. Another review further noted that Solazyme developed a nutritional formulation based on Porphyridium polysaccharides aimed at reducing inflammation and oxidative damage [246].

### 8.5. Research Gap

Compared with *spirulina*-based systems, direct human evidence for purified red seaweed PBPs remains limited. Systematic reviews and meta-analyses have quantified changes in inflammation and oxidative stress markers at the population level for spirulina supplements, whereas such evidence is considerably more limited for R-PE or APC derived from red seaweed [150]. Existing reviews of PBPs largely summarize health effects based on in vitro and animal studies, and further highlight that the mechanistic understanding and evidence chain require strengthening [247]. This also indicates that more robust clinical validation is necessary before extrapolating these findings to human populations.

From a regulatory perspective, coordination and standardization of specifications remain significant barriers to global expansion. The FDA approval of *Galdieria* extract blue via the color additive petition pathway reflects the increasing regulatory acceptance of seaweed-derived natural colorants [248]. At the same time, this also underscores that such approvals are based on clearly defined identity, specifications, and intended use, and are therefore limited to specific raw materials and use conditions.

## 9. Conclusions

PBPs from red seaweeds occupy a unique position within the field of natural pigments. On one hand, they offer clear technical advantages, including vivid color performance, water solubility, and relevance to clean-label product development. On the other hand, their value extends beyond color functionality alone. The literature also supports their potential as multifunctional ingredients, particularly with respect to antioxidant, anti-inflammatory, antibacterial, and other bioactive properties. For this reason, red algal PBPs are better understood not merely as alternative pigments, but as candidate ingredients for dual-function food design.

However, several obstacles continue to limit the widespread application of PBPs. Variations in species, cultivation environments, and harvesting conditions constrain upstream development by affecting pigment composition and batch-to-batch consistency. In addition, the high cost and limited scalability of conventional extraction and purification methods remain significant challenges. Although emerging extraction and purification technologies are developing rapidly, stability and safety still represent major barriers. Due to the high sensitivity of PBPs to heat, light, oxygen, and pH, appropriate encapsulation and storage strategies also require further investigation. From a health perspective, current evidence is still largely derived from in vitro and animal studies, predominantly within *Spirulina*-based systems. Accordingly, findings related to purified red algal PBPs should be interpreted with appropriate caution. Stronger clinical validation, clearer dose standardization, and more robust assessment of bioavailability and safety are necessary to translate their potential into practical applications.

Overall, current evidence does not yet demonstrate that red algal PBPs can fully replace conventional pigment systems. However, it does highlight the practical value of these pigments, providing a credible conceptual direction for future clean-label color design and health-oriented food innovation. Progress in this field will no longer rely on repeatedly emphasizing potential alone, but will require improved standardization of raw materials, more robust stabilization strategies, scalable and food-grade processing technologies, stronger clinical validation, and clearer regulatory pathways. With these advances, red algal PBPs may evolve from niche pigments into multifunctional ingredients with commercial relevance, supporting both clean-label coloring applications and future health-oriented food system design.

## Figures and Tables

**Table 1:** Nomenclature of major phycobiliprotein (PBP) types and subtypes discussed in this review.

Category	Subtype	English Designation	Main Distinction	Reference
PE	R-PE	R-phycoerythrin	R denotes Rhodophytan origin; common red algal PE	[14,21]
	B-PE	B-phycoerythrin	B denotes Bangiophycean origin	
	C-PE	C-phycoerythrin	C denotes cyanobacterial origin	
	C-PE-I C-PE-II	C-phycoerythrin I C-phycoerythrin II	Mainly PEB-containing C-PE type	[20,21]
			Contains PUB in addition to PEB; associated with stronger blue-green light absorption in some marine cyanobacteria	
PC	R-PC	R-phycocyanin	R denotes Rhodophytan origin; includes several reported R-PC subtypes	[21,22]
	R-PC(I)	R-phycocyanin I	PCB at α84 and β84; PEB at β155	[22,23]
	R-PC(II)	R-phycocyanin II	PEB at α84 and β155; PCB at β84	[23]
	R-PC(III)	R-phycocyanin III	Reported as an R-PC subtype with chromophores at α84, β84, and β155; exact bilin assignment is less consistently summarized	[21]
	R-PC(IV)	R-phycocyanin IV	Differs from R-PC(I)–R-PC(III) in chromophore-binding pattern; associated with PUB-containing α-subunit chromophores	[21]
	R-PC(V)	R-phycocyanin V	Trichromatic phycocyanin carrying PUB on the α subunit, particularly at αCys84	[24]
	C-PC	C-phycocyanin	C denotes cyanobacterial origin; commonly contains PCB as the major chromophore	[21]
APC	APC	Allophycocyanin	Conserved PBS core PBP; APC-B and APC-Lcm are APC-related core components	[21,25]
PEC	PEC	Phycoerythrocyanin	Sometimes proposed as a fourth PBP class; spectrally intermediate between PE and PC	[14,21]

Abbreviations: PBP, phycobiliprotein; PBS, phycobilisome; PE, phycoerythrin; PC, phycocyanin; APC, allophycocyanin; PEC, phycoerythrocyanin; PEB, phycoerythrobilin; PCB, phycocyanobilin; PUB, phycourobilin.

**Table 2:** Comparison of red seaweed species, pigment yields, and purity indices.

Species	Target Pigment	Yield	Purification Method	Purity Index	Purity Value	Reference
*Gracilaria gracilis*	R-PE	1.26 mg g^−1^ dw	Anion-exchange chromatography	A565/A280	3.25	[41]
*Gracilaria chilensis*	R-PE	0.20 mg g^−1^ dw	Ammonium sulfate precipitation Anion-exchange chromatography	A566/A280	2.18 ± 0.11	[42]
	R-PC	0.23 mg g^−1^ dw		A620/A280	2.04 ± 0.09	
*Gracilariopsis lemaneiformis*	R-PE	0.5 mg R-PE/100 mg crude sample	Ammonium sulfate precipitation Centrifugal precipitation chromatography	A565/A280	6.5	[43]
*Neopyropia yezoensis (Pyropia yezoensis)*	R-PE	1.5 mg g^−1^ dw	Ammonium sulfate precipitation Fast protein liquid chromatography	A565/A280	5.4	[35]
*Grateloupia turuturu*	R-PE	5.28 mg g^−1^ dw	Anion-exchange chromatography	A565/A280	2.89	[44]
*Sarcopeltis skottsbergii*	R-PE	5.67 ± 0.42 mg g^−1^ dw	High-pressure homogenization	A565/A280	1.57 ± 0.01	[45]
		2.17 ± 0.10 mg g^−1^ dw	Ultrasound-assisted extraction		1.15 ± 0.02	
*Solieria filiformis*	R-PE	0.14 mg g^−1^ wet seaweed 0.02 mg g^−1^ wet seaweed (purified R-PESf)	Ammonium sulfate precipitation Anion-exchange chromatography Size exclusion chromatography	A564/A280	4.50	[46]
*Kappaphycus alvarezii*	PE	Crude extract: 78.43 µg mL^−1^ final: 554.12 µg mL^−1^	Ammonium sulfate precipitation Gel permeation chromatography	A620/A280	5.63	[47]

Abbreviations: PE, phycoerythrin; R-PE, R-phycoerythrin; R-PC, R-phycocyanin. dw denotes dry weight. Purity index is expressed as Aλ/A280, where A denotes absorbance and λ denotes the target PBP absorption wavelength.

**Table 3:** Summary of major extraction methods for PBPs.

Method	Main Role	Advantages	Limitations	References
Conventional cell disruption, including freeze–thaw, liquid-nitrogen-assisted grinding, and bead milling	Disrupt algal cells or tissues and release intracellular PBPs into the aqueous phase	Technologically mature; simple to operate; bead milling is relatively scalable; can be conducted under mild, cold, and light-protected conditions	Dense red algal tissues and polysaccharide-rich matrices may reduce disruption efficiency, increase energy demand, and interfere with downstream clarification or chromatographic separation	[32,42,43,56,61,62,63]
Enzyme-assisted extraction (EAE)	Degrade algal cell-wall components and enhance the release of water-soluble PBPs	Mild; can improve extraction efficiency; enzyme mixtures may further enhance recovery depending on biomass type	High enzyme cost, limited reusability, and substrate specificity remain limitations	[83,84,85,86]
Ultrasound-assisted extraction (UAE)	Enhance cell disruption and mass transfer through acoustic cavitation	Rapid; relatively energy-efficient; can increase target protein yield and shorten extraction time	Ultrasonic power and operating conditions may induce localized hotspots, potentially degrading polysaccharides or target proteins; large-scale applications remain limited	[82,83,87,99]
Microwave-assisted extraction (MAE)	Accelerate cell disruption and extraction through microwave-induced heating	Very rapid; can greatly reduce extraction time compared with conventional soaking	Heat accumulation may reduce PE yield and cause protein denaturation or chromophore bleaching; careful temperature control is required	[82,83,88,89,90]
Pressurized water and pressurized fluid extraction	Enhance solubility, mass transfer, and protein release under pressurized conditions	Efficient, scalable, and attractive for industrial application	May co-extract non-target compounds, which need to be addressed during downstream purification	[82,91,92]
DES/NADES-based extraction	Improve pigment recovery by disrupting biomass matrices through hydrogen-bonding solvent systems	Low volatility and low toxicity; may improve extraction efficiency and stabilize protein structures to some extent	Food-grade use still requires further validation regarding solvent recovery or retention, metabolic fate, and regulatory approval	[64,94,95,96,97,98,100]

**Table 4:** Applications of Red Algal Phycobiliproteins in Different Food Systems.

Food System	Representative PBP and Source	Primary Role in Formulation	Key Outcome	References
Beverages	PE (*Porphyridium cruentum*)	Natural colorant	Effective coloring at low dose; stable pink color during chilled storage; good sensory acceptance	[15,109]
Dairy products	B-PE (*Porphyridium purpureum*)	Natural pink colorant	B-PE extract achieved pink coloration in milk-based products at low colorant levels and showed good stability across pH 4.0–9.0.	[108]
Fermented dairy products	PE (*Gracilaria gracilis*); PE/PC (*Nostoc* sp.; *Spirulina* sp.)	Natural colorant; functional enrichment	Applied in yogurt, low-fat yogurt, and cream cheese. Red algal PBPs supported coloration in yogurt, while cyanobacterial PE/PC contributed antioxidant and antimicrobial effects during refrigerated storage.	[129,130]
Confectionery	R-PE/PE (red seaweeds)	Natural colorant	Potential application in jellies, chewing gums, and sweetened gel systems, although direct application evidence remains limited	[15,107,110]
Meat analogues	R-PE (*Furcellaria lumbricalis*)	Natural colorant	Meat-like pink color before frying and brown surface after frying, supporting use in plant-based meat products	[110,133]

Abbreviations: PE, phycoerythrin; B-PE, B-phycoerythrin; R-PE, R-phycoerythrin; PBP, phycobiliprotein. Evidence for confectionery applications is currently based mainly on review-level summaries. Cyanobacterial PE/PC studies are included as supportive evidence to illustrate the functional potential of PBPs in dairy matrices, as direct red algal evidence remains limited.

**Table 5:** Comparison of Major Natural Colorant Classes and Their Application Constraints.

Category	Main Hue and Phase	Key Advantages	Key Limitations	Best-Suited Products/Processes
Anthocyanins	Red-purple; aqueous systems	Broad hue range; strong color	pH-dependent hue shift; sensitive to light/heat/O_2_/metal ions; weak near neutral pH [4,137,139]	Acidic beverages; acidic dairy; confectionery
Betalains	Red-violet (betacyanins), yellow-orange (betaxanthins); aqueous systems	Vivid red; clean-label	Sensitive to heat/light/O_2_/water activity; shelf-life challenges [140,141]	Cold/mild processing; beverages; desserts
Carotenoids	Yellow-orange-red; mostly lipophilic	Strong hues; mature ingredient options	Oxidative/light-induced degradation; often requiring encapsulation or delivery systems; losses under strong light/O_2_ [4,137,142]	Fat-containing or emulsified foods
Chlorophylls and derivatives (E140/E141)	Green; native mostly lipophilic; derivatives more dispersible	Iconic green; E141 more stable	Native chlorophyll readily loses green under acidic conditions; light/heat further accelerate degradation, whereas copper derivatives are more stable [4,107,137,143]	Products needing stable green (often via E141)
PBPs	Blue (C-PC), red-pink (PE); aqueous systems	Rare clean-label blue/pink; strong water-phase color	Denaturation/photo-oxidation; heat and pH sensitive (especially pH < 5) (acidic systems challenging) [107,114,144]	Cold/mild processing; post-process addition

Abbreviations: PBP = phycobiliproteins; C-PC = C-phycocyanin; PE = phycoerythrin; E140/E141 = chlorophylls and copper chlorophyll derivatives.

**Table 6:** Biological activities of PBPs and supporting evidence.

Activity	Mechanism	Model System	Key Finding	References
Antioxidant	Radical scavenging, reducing power, and metal chelation	Red seaweed-derived PE/PBP; ABTS, FRAP, DPPH, and FIC assays	Red seaweed PBPs showed measurable in vitro antioxidant activity	[35,47,174,175]
Anti-inflammatory	Suppression of inflammatory mediators	Red algae-derived PE/peptides; enzyme, cell, and animal models	Showed anti-inflammatory potential in vitro (COX-2 inhibition and reduced inflammatory cytokines)	[153,176,177]
Anticancer	Growth inhibition and apoptosis induction	Red algae-derived PE/PBPs; cancer cell models	Showed anticancer activity in vitro by reducing cell viability and inducing apoptosis	[47,158,175,178]
Antimicrobial	Growth inhibition of foodborne or clinical pathogens	Red seaweed-derived PE; in vitro antimicrobial assays	Showed measurable antimicrobial activity against selected bacteria	[47,174,178]
Metabolic health	Modulation of lipid metabolism and ACE inhibition	Red algae-derived PE/peptides; adipocyte, hepatocyte, and in vitro ACE assays	Showed potential to regulate lipid metabolism and support cardiometabolic health in preclinical models	[179,180,181]

Abbreviations: PE, phycoerythrin; PBP, phycobiliprotein; ABTS, 2,2′-azinobis-(3-ethylbenzothiazoline-6-sulfonic acid) assay; FRAP, ferric reducing antioxidant power; DPPH, 2,2-diphenyl-1-picrylhydrazyl assay; FIC, ferrous ion-chelating assay; ACE, angiotensin-converting enzyme.

## Data Availability

No new data were created or analyzed in this study. Data sharing is not applicable to this article.
